# Apicoplast-Localized Lysophosphatidic Acid Precursor Assembly Is Required for Bulk Phospholipid Synthesis in *Toxoplasma gondii* and Relies on an Algal/Plant-Like Glycerol 3-Phosphate Acyltransferase

**DOI:** 10.1371/journal.ppat.1005765

**Published:** 2016-08-04

**Authors:** Souad Amiar, James I. MacRae, Damien L. Callahan, David Dubois, Giel G. van Dooren, Melanie J. Shears, Marie-France Cesbron-Delauw, Eric Maréchal, Malcolm J. McConville, Geoffrey I. McFadden, Yoshiki Yamaryo-Botté, Cyrille Y. Botté

**Affiliations:** 1 ApicoLipid group, Institute for Advanced Biosciences UMR5309, CNRS, Université Grenoble Alpes, INSERM, Grenoble, France; 2 The Francis Crick Institute, The Ridgeway, Mill Hill, London, United Kingdom; 3 Department of Biochemistry and Molecular Biology, Bio21 Molecular Science & Biotechnology Institute, University of Melbourne, Melbourne, Victoria, Australia; 4 Centre for Chemistry and Biotechnology, School of Life and Environmental Sciences, Deakin University, Burwood, Victoria, Australia; 5 School of BioSciences, University of Melbourne, Melbourne, Victoria, Australia; 6 Research School of Biology, Australian National University, Canberra, Australian Capital Territory, Australia; 7 BNI group, TIMC-IMAG UMR5525 CNRS, Université Grenoble Alpes, Grenoble, France; 8 Unité de recherche (UMR) 5168, CNRS, CEA, INRA, Université Grenoble Alpes, Grenoble, France; Albert Einstein College of Medicine, UNITED STATES

## Abstract

Most apicomplexan parasites possess a non-photosynthetic plastid (the apicoplast), which harbors enzymes for a number of metabolic pathways, including a prokaryotic type II fatty acid synthesis (FASII) pathway. In *Toxoplasma gondii*, the causative agent of toxoplasmosis, the FASII pathway is essential for parasite growth and infectivity. However, little is known about the fate of fatty acids synthesized by FASII. In this study, we have investigated the function of a plant-like glycerol 3-phosphate acyltransferase (*Tg*ATS1) that localizes to the *T*. *gondii* apicoplast. Knock-down of *TgATS1* resulted in significantly reduced incorporation of FASII-synthesized fatty acids into phosphatidic acid and downstream phospholipids and a severe defect in intracellular parasite replication and survival. Lipidomic analysis demonstrated that lipid precursors are made in, and exported from, the apicoplast for *de novo* biosynthesis of bulk phospholipids. This study reveals that the apicoplast-located FASII and ATS1, which are primarily used to generate plastid galactolipids in plants and algae, instead generate bulk phospholipids for membrane biogenesis in *T*. *gondii*.

## Author Summary

Apicomplexan parasites are the causative agents of several major human diseases including toxoplasmosis and malaria, caused by *Toxoplasma gondii* and *Plasmodium* spp. respectively. Obligate intracellular stages of these parasites undergo periods of rapid asexual replication which require synthesis of large amounts of lipids for membrane biogenesis. These lipids can either be scavenged from the host cell and/or synthesized *de novo* by the parasite. Many of the enzymes involved in *de novo* fatty acid biosynthesis are localized in a non-photosynthetic relict plastid, named the apicoplast, suggesting that this organelle may generate fatty acids and phospholipids that are used for bulk membrane biogenesis. However, it is not known to what extent fatty acids generated by the apicoplast are exported to other cell membranes. Here we show that a key enzyme required for bulk phospholipid biosynthesis, glycerol 3-phosphate acyltransferase, is localized to the *T*. *gondii* apicoplast. Comprehensive lipidomic analysis of an acyltransferase null mutant suggested that apicoplast-synthesized phospholipid precursors are subsequently exported and used for bulk phospholipid synthesis. This process is essential for parasite growth and virulence in host cells.

## Introduction

Apicomplexan parasites include the etiological agents of a number of devastating human diseases, including malaria (*Plasmodium* spp.), toxoplasmosis (*Toxoplasma gondii*) and cryptosporidiosis (*Cryptosporidium* spp.). Most Apicomplexa harbor a plastid of prokaryotic origin, termed the apicoplast [1–3]. While the apicoplast lacks the enzymes involved in photosynthesis, this organelle contains many of the other metabolic pathways found in plant and algal plastids, including a prokaryotic type II fatty acid synthesis pathway (FASII) [4]. Since the apicoplast is essential for parasite survival, some of these pathways are considered attractive drug targets [5–8]. The discovery of the FASII pathway suggested that these parasites synthesise fatty acids (FA) *de novo*, rather than relying solely on supply from their host as was initially thought [4, 9, 10]. Subsequent genetic studies showed that components of the *T*. *gondii* FASII pathway (e.g. acyl carrier protein) are essential for the intracellular growth of the rapidly dividing tachyzoite stages [11, 12]. FASII is also essential to parasite development in mosquito stages of *P*. *falciparum* [13] and liver stages of *P*. *berghei* and *P*. *yoelii*, the rodent malaria models [14, 15].

Upon invasion of host cells, most apicomplexan parasites become surrounded by a membrane, termed the parasitophorous vacuolar membrane (PVM), which expands as the parasite develops and replicates. Both PVM expansion and parasite plasma/organelle membrane production during replication are reliant on recruitment of phospholipids (PLs) for membrane biogenesis. Indeed, PLs are the major lipid class found in *T*. *gondii* parasite membranes, accounting for up to 80–90% of the total lipid content [16, 17]. PL synthesis is essential for parasite replication and enzymes involved in their assembly are promising drug targets [18–20]. In *T*. *gondii*, PL assembly is supported by both scavenged and *de novo*-synthesised FA, while mature PLs such as phosphatidylcholine (PC) may also be scavenged [21]. PC is the most abundant PL species in *T*. *gondii* and *P*. *falciparum* membranes [16, 17]. PC synthesis is critical for *T*. *gondii* tachyzoite replication and *Plasmodium* blood and liver stages [22–25], while specific inhibitors of PC biosynthesis are currently in clinical trials as anti-malarial drugs [26, 27]. Other important PLs include phosphatidylethanolamine (PE), accounting for up to 20% PL in these parasites [16, 17], and phosphatidylinositol (PI), which is essential for apicoplast biogenesis, glycolipids, glycosylphosphatidylinositol anchors (GPI), membrane dynamics and integrity, and parasite survival [17, 28–31].

PL synthesis is initiated by successive acyltransferase-dependent additions of fatty acyl chains to the hydroxyl groups of glycerol 3-phosphate (G3P) to generate phosphatidic acid (PA). PA is the central precursor for the *de novo* synthesis of diacylglycerol by phosphatidic acid phosphatase (PAP) and for the synthesis of CDP-dicacylglycerol (CDP-DAG) by CDP-DAG synthase (CDS) [32, 33]. In Apicomplexa, as in algae/plants, most modification of FA (i.e. elongation, dehydration) and major steps for *de novo* synthesis of PL using PA as central precursor occur in the endoplasmic reticulum (ER). However, the exact details of PL synthesis are not yet fully understood and apparently involve other organelles such as mitochondria [33–35]. Moreover, the source of acyl chains required for PA assembly in apicomplexan parasites remains unclear. Plants have two pathways for *de novo* PA assembly, namely an ER-localised ‘eukaryotic-origin’ pathway and a plastid-localised ‘prokaryotic-origin’ pathway, which produce distinct PL products [33]. The ER-localised pathway generates PC and galactolipids (which are ultimately trafficked to the chloroplast), while the plastid pathway generates galactolipids, sulfolipids, and phosphatidylglycerol (PG) [33]. Both sources of galactolipids are essential for chloroplast structural integrity and photosynthetic function in plants and, in phosphate-deprived conditions, can replace PL in extra-plastidial membranes (for reviews, see [36–38]). Plastid PA is synthesized *de novo* by the initial esterification of an acyl chain onto the *sn*-1 position of G3P by a G3P acyltransferase (G3PAT, or ATS1), followed by esterification of a second acyl chain onto the *sn*-2 position by an acyl-G3P acyltransferase (LPAAT, or ATS2) [32].

While Apicomplexa lack galactolipids [17, 39, 40], genome mining of *T*. *gondii* and *Plasmodium* spp. reveals putative homologs of *ATS1* and *ATS2* [10, 41–43]. Interestingly, a recent study on *P*. *yoelii* showed that a homolog of ATS1 (*Py*apiG3PAT) was essential for the development of late liver stages, phenocopying *P*. *yoelii* and *P*. *bergheii* FASII mutants [44]. However, the precise role of the apicoplast in parasite membrane biogenesis and the intracellular fate of plastid-synthesised FA are unknown in both *T*. *gondii* and *Plasmodium*. Whilst there is evidence for the ‘eukaryotic-origin’ pathway in *P*. *falciparum* since it possesses an ER membrane-bound G3PAT, the function of this enzyme is still unknown [45]. Moreover, an ER membrane-bound G3PAT is yet to be characterized in *T*. *gondii*.

There is considerable complexity in the acyl chains incorporated into PA and PL. Apicoplast-localised FASII generates FA up to a chain length of 14–18 carbons, which can be exported to the ER for further elongation [12, 17, 46]. How and where these FA are incorporated into PA is not known. A better understanding of PA assembly, and how its downstream products are then distributed throughout the parasite membrane network, will be crucial in fully understanding membrane biogenesis in these parasites.

To determine the apicoplast contribution to PL biosynthesis in *T*. *gondii*, we generated a conditional *TgATS1* mutant. We found that *Tg*ATS1 is targeted to the apicoplast and is critical for organelle formation, parasite growth and normal intracellular development. ^13^C-glucose metabolic labelling and mass spectrometry-based lipidomic analyses revealed major defects in incorporation of apicoplast-synthesized C14:0 and PL assembly in *Tg*ATS1-deficient parasites. Our results show that the apicoplast *Tg*ATS1 is responsible for the synthesis of a C14:0-containing lysophosphatidic acid (LPA, the obligate intermediate in PA production), which is subsequently used to assemble major PL classes (PC, PE and PI).

## Results

### *Tg*ATS1 is a plastid-localised algal/plant-like glycerol 3-phosphate acyltransferase

**Fig 1:**
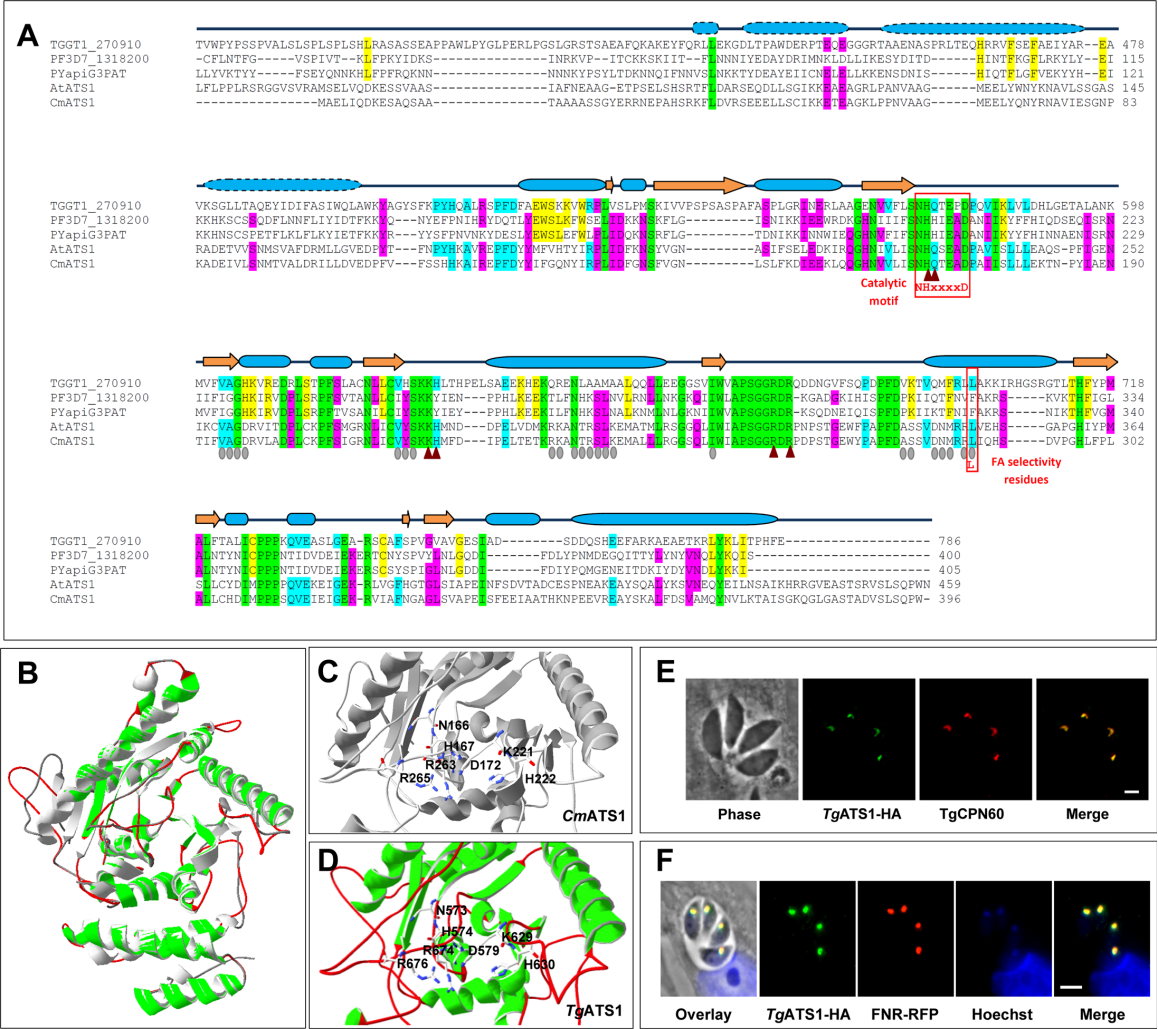
*Tg*ATS1 is a plant-like G3PAT residing in the stroma of the *T*. *gondii* apicoplast. **(A)** Structure-based protein sequence alignment of G3PATs from *T*. *gondii*, *Tg*ATS1 (TGGT1_270910), *P*. *falciparum* (PF3D7_1318200), *P*. *yoelii* (*Py*apiG3PAT), *A*. *thaliana* (*At*ATS1, [68]) and *C*. *moschata* (*Cm*ATS1, [47]). Residues strictly conserved between all species are highlighted in green, residues conserved in at least three species in cyan, residues conserved in apicomplexan sequences in yellow, and residues conserved between *Tg*ATS1 and plant ATS1 (*At*ATS1 and *Cm*ATS1) in blue. Brown triangles and grey ovals represent residues putatively involved in G3P or FA binding, respectively. Secondary structures [47, 67] are represented above the sequence alignment by blue cylinders for α-helices and orange arrows for β-strands. Domain 1 of the protein (4-helix bundle) is symbolized with dashed lines and Domain 2 (α-β Domain) in solid lines. Residues putatively involved in binding the G3P substrate in *Cm*ATS1 (brown triangles) are strictly conserved in *Tg*ATS1, while those putatively involved in binding the acyl-ACP substrate are highly conserved (grey ovals). **(B)** Overlay of the *Cm*ATS1 crystal structure ([47]) and the predicted *Tg*ATS1 3D structure. The overall structure and surface accessibility of *Cm*ATS1 (grey) and *Tg*ATS1 (green and magenta) is conserved and highly similar as observed in the ribbon representation. **(C)** Residues putatively involved in binding substrate (G3P) and those involved in the catalytic motif NHX_4_D of *Cm*ATS1 form a catalytic pocket with His-167 and Asp-172. **(D)** Both the motif and topology of the pocket are strictly conserved in *Tg*ATS1 (His-574 and Asp-579). **(E, F)** IFA shows that *Tg*ATS1 is a stromal-resident protein of the apicoplast, as confirmed by co-localization with fluorescence of **(E)** the chimeric apicoplast stromal FNR-RFP reporter protein co-expressed in the *TgATS1*-iKO parasite line and **(F)** anti-CPN60, a known marker of the apicoplast stroma [51]. Scale bars represent 2 μm.

In plant and algal plastids, PA biosynthesis is initiated by a soluble G3PAT called ATS1. Searches of the *T*. *gondii* genome with plant ATS1 genes revealed a homolog that we named *T*. *gondii* acyl transferase 1 (*TgATS1*) (Fig 1A). Structure modelling of *Tg*ATS1 using the *Cucurbita moschata* (squash or pumpkin) ATS1 (*Cm*ATS1) structure [47, 48] as a threading template suggests that *Tg*ATS1 contains two domains: one comprising a four α-helix bundle, and a second larger domain formed of 11 α-helices and 10 mixed parallel/anti-parallel β-sheets (Fig 1A–1D). The larger domain apparently contains the substrate-binding and catalytic sites involved in (i) G3P binding, (ii) FA binding, (iii) catalysis (NHX_4_D motif), and (iv) FA selectivity, similar to the plant ATS1 (Fig 1A and 1B). The NHX_4_D motif typical of all G3PATs [49] is localised to the same putative groove in *Cm*ATS1 (Fig 1C, detailed in S1A–S1C Fig) and *Tg*ATS1 (Fig 1D, detailed in S1D–S1F Fig). Furthermore, the cluster of positively charged residues that surround the catalytic pocket and bind G3P in *Cm*ATS1 (Fig 1C; H167, K221, H222, R263, R265) are conserved in *Tg*ATS1 (Fig 1D). Plastid ATS1s are soluble whereas non-plastid G3PATs are membrane-bound [45]. *Tg*ATS1 has no obvious transmembrane domains, suggesting it is also soluble. Phylogenetic analysis confirms that *Tg*ATS1 clusters with algal, plant, and photosynthetic ATS1s and diverges from eukaryotic GPATs (S1G Fig)

*Tg*ATS1 is predicted to localize to the apicoplast due to the presence of a bipartite N-terminal targeting sequence [41, 50]. To localize *Tg*ATS1, we generated a construct that expressed *TgATS1* with a C-terminal triple haemagglutinin (3×HA) epitope-tag under control of an anhydrotetracycline (ATc)-regulated promoter [51] and expressed this construct in parasites expressing the apicoplast marker ferredoxin NADP^+^ oxidoreductase-red fluorescent protein (FNR-RFP) (S2A Fig). Immunofluorescence assays (IFA) co-localized *Tg*ATS1-HA protein with FNR-RFP (Fig 1E) and another apicoplast stromal marker, chaperonin 60 (CPN60) (Fig 1F). Taken together, these data indicate that *Tg*ATS1 is a plant-like, apicoplast-localised homolog of ATS1.

### Disruption of *TgATS1* causes defects in organellar and daughter cell development

To investigate the role of *Tg*ATS1 in parasite growth, we disrupted the endogenous copy of *TgATS1* (*eTgATS1*) by insertion of a selectable marker (chloramphenicol acetyl transferase, CAT) into the *TgATS1* ORF using a double recombination approach with a recombineering cosmid (S2B Fig, [52]). Four independent *TgATS1-HA*-iKO strains were generated: two bearing a TATi-inducible *Tg*ATS1-HA copy (*TgATS1-HA*-iKO), and two equivalent mutants in the *FNR-RFP* background (*TgATS1-*iKO/*FNR-RFP*). Disruption of the endogenous gene locus was confirmed by PCR (S2C Fig) and Southern blotting (S2D Fig). All subsequent analyses were independently performed using each mutant *TgATS1* line.

**Fig 2:**
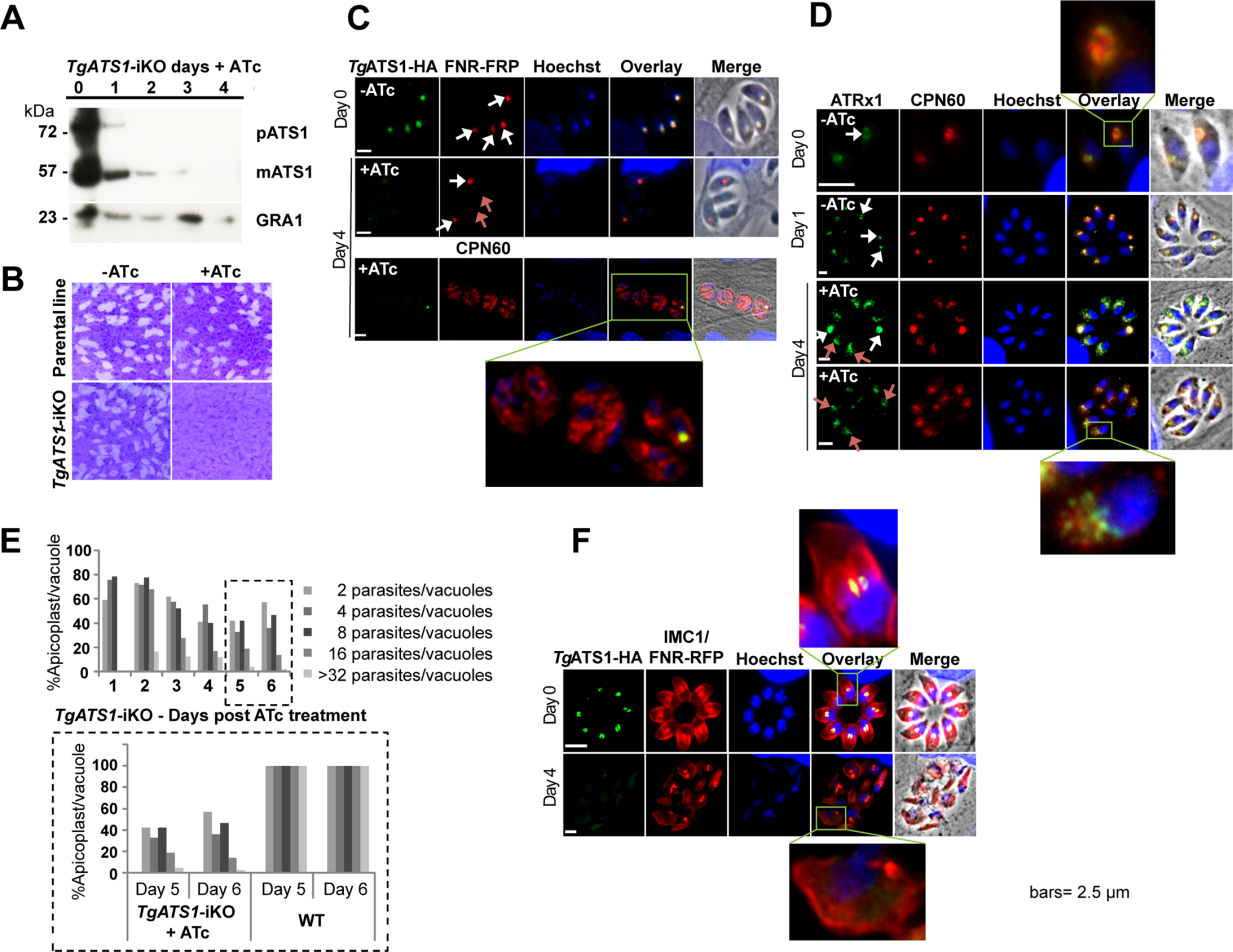
*Tg*ATS1 is critical for normal intracellular development and division. **(A)** Inducible knockdown of *Tg*ATS1 in the *TgATS1-HA*-iKO line. *Tg*ATS1 was detected by Western blot analysis using anti-HA antibody as two bands: the pre-mature form (pATS1, ~75KDa) and the mature form (mATS1, ~55KDa). Protein expression was down-regulated to undetectable levels by 3 or 4 days of ATc treatment (1 mg/mL ATc, numbers indicate days of culture). GRA1 (lower panel) served as a loading control. **(B)** Plaque assays performed in the absence (-) or presence (+) of ATc and fixed after 10 days show an impaired lytic cycle of *TgATS1-HA*-iKO parasites in the presence of ATc. **(C)** IFA of *TgATS1-HA*-iKO parasites using anti-HA antibody and apicoplast stromal markers FNR-RFP (two upper panels) and CPN60 antibody (lower panel) indicates loss of HA and apicoplast signals in the presence of ATc for 4 days (white arrows indicate normal apicoplast while red arrows indicate loss of apicoplast signal), as well as the cytosolic mis-localisation of apicoplast CPN60 (lower panel, zoomed area). **(D)** IFA of *TgATS1-HA*-iKO parasites using antibodies against the apicoplast stromal marker CPN60 and the apicoplast outer membrane marker ATRx1 confirms mis-localisation of CPN60 and ATRx1, indicating loss of apicoplast structure (lower panels, zoomed areas). White arrows indicate normal apicoplast and red arrows indicate a normal apicoplast. **(E)** Quantification of the number of intact apicoplasts relative to parasites and vacuoles in *TgATS1-HA*-iKO parasites following ATc treatment. A significant loss of apicoplasts was observed in the presence of ATc (upper graph) of up to 60% at days 5 and 6, compared to the wild type (parental) strain that contained 100% apicoplasts in all vacuoles regardless of the number of parasites per vacuole (lower panel). (n = 100 vacuoles). **(F)** IFA of *TgATS1-HA*-iKO parasites using anti-IMC1 antibody grown in the presence and absence of ATc indicates IMC structure defect (zoomed areas). Scale bars: 2.5 μm.

*Tg*ATS1-HA was detected by Western blot as two distinct bands with apparent molecular masses of 72 kDa and 55 kDa (Fig 2A). These likely correspond to the pre-processed (pATS1, including the complete or partial N-terminal bipartite sequence) and mature (mATS1, apicoplast-resident) forms of the protein, respectively. Addition of ATc to the culture medium down-regulated *TgATS1-HA*-iKO expression, with pATS1 and mATS1 no longer detectable by Western blot after 3 and 4 days, respectively (Fig 2A). Plaque assays were performed to determine the effect of *TgATS1* down-regulation on parasite growth. ATc treatment of parental lines produced no detectable growth defect (Fig 2B, top panels). ATc treatment of *TgATS1-HA-*iKO parasites resulted in almost complete ablation of host cell lysis and plaque formation (Fig 2B, bottom right panel), although growth assays after 8 days of *TgATS1* repression in a *TgATS1-HA-*iKO strain expressing cytosolic tdTomato indicated that some parasites may continue to grow, albeit slowly (S3 Fig).

To discern the cause of this growth arrest, we examined mutant parasites by IFA. Strikingly, we observed partial loss of apicoplast-localized FNR-RFP after 4 days of ATc treatment, with some parasites displaying no signal at all (Fig 2C). IFA against the apicoplast stromal protein, CPN60, revealed that loss of *Tg*ATS1 resulted in redistribution of CPN60 from a typically punctate apicoplast staining to a diffuse cytoplasmic staining (Fig 2C). This was also observed using antibodies against the apicoplast outer membrane marker ATRx1 (Fig 2D), suggesting a loss of apicoplast structure. To investigate this further we quantified the number of parasites containing an intact apicoplast over the course of 6 days of ATc treatment (Fig 2E). Although some parasites lost their apicoplast within the first day, the greatest loss appeared after 5–6 days of ATc treatment. Inner membrane complex (IMC) formation (as observed with anti-IMC antibodies) was also greatly affected and, together with apicoplast abrogation, was associated with changes in parasite morphology (Fig 2F). These observations suggested that *Tg*ATS1 is required for organelle formation and parasite division.

**Fig 3:**
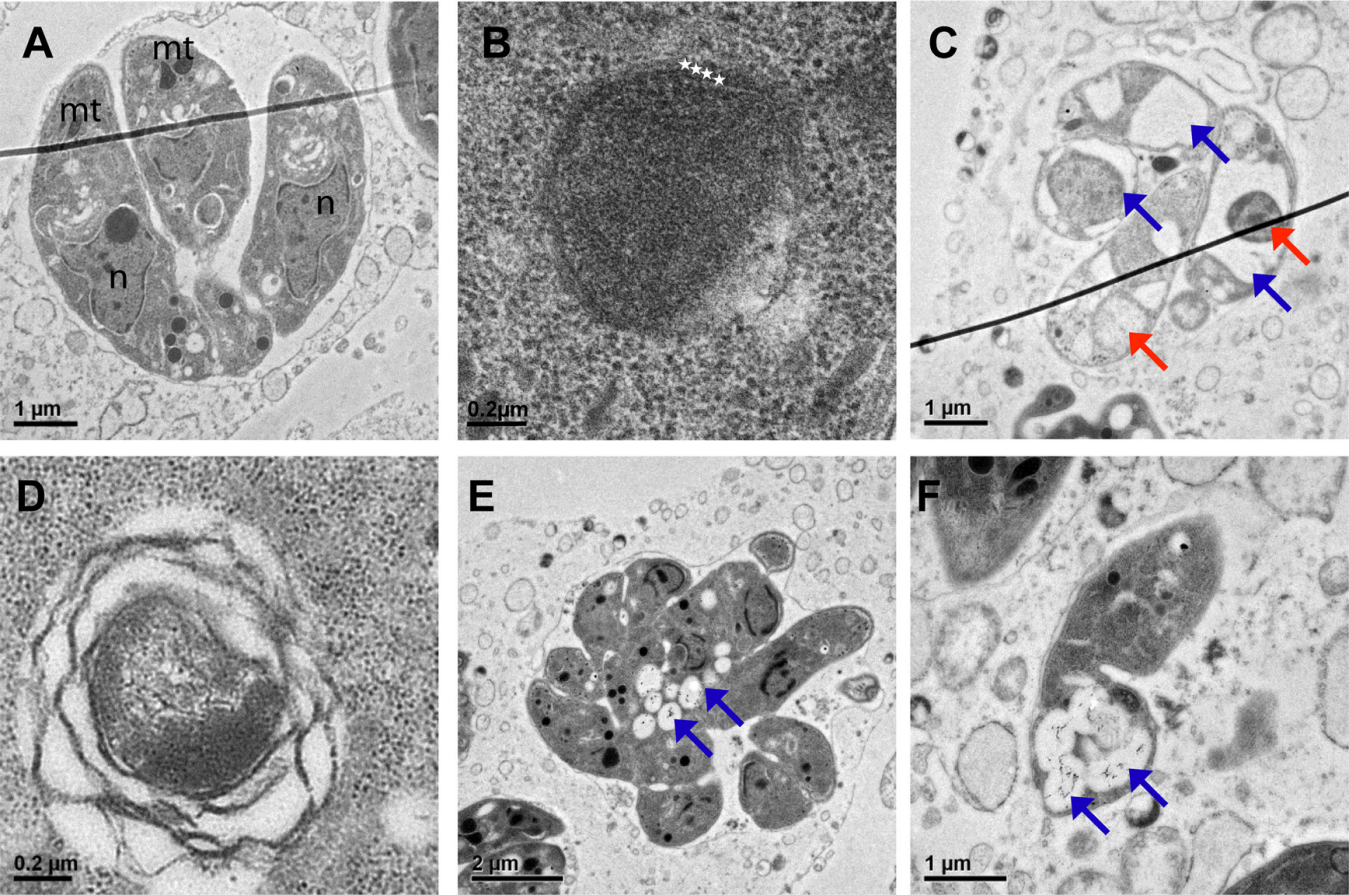
*TgATS1* disruption affects tachyzoite division and the morphology of intracellular organelles. Transmission electron micrographs showing a typical vacuole containing 4 *TgATS1*-iKO intracellular tachyzoites in the absence of ATc, each bearing normal intracellular organelles such as the mitochondrion (mt) and nucleus (n) **(A)**, with an apicoplast surrounded by 4 membranes as indicated by white stars **(B)**. In the presence of ATc, intracellular development of *TgATS*-iKO parasites was drastically affected, resulting in parasites bearing aberrant organelles shown by red arrows and large electron lucent regions shown by blue arrows **(C)**. Apicoplast biogenesis was also affected in the presence of ATc, with only a few parasites bearing an apicoplast and, of those present, morphological aberrations were observed, including disorganized membranes and atypical stroma **(D)**. Intracellular division also seemed affected upon *Tg*ATS1 disruption **(E)**. Parasites often displayed large electron-lucent vesicles containing an unusual ribbon-like material shown by blue arrows **(E, F).** Scale bars are indicated in each figure.

Transmission electron microscopy of *TgATS1-*iKO parasites revealed further defects in intracellular parasite morphology (Fig 3). Untreated parasites morphology was unperturbed (Fig 3A), containing apicoplasts with the canonical four membranes (Fig 3B). However, after 3 days of ATc treatment, a number of ultrastructural changes were observed including (i) the accumulation of large electron-lucent vesicles and abnormal intracellular compartments (Fig 3C); (ii) the appearance of apicoplasts with grossly malformed membranes that had detached from each other (Fig 3D); (iii) the complete loss of apicoplasts (Fig 3E); (iv) and the accumulation of multiple vacuoles harbouring filamentous material (Fig 3F). Taken together, these studies show that loss of *TgATS1* expression leads to marked changes in apicoplast morphology and defects in the biogenesis of this organelle and parasite development.

### *Tg*ATS1 disruption reduces FASII C14:0 generation and FA elongation

**Fig 4:**
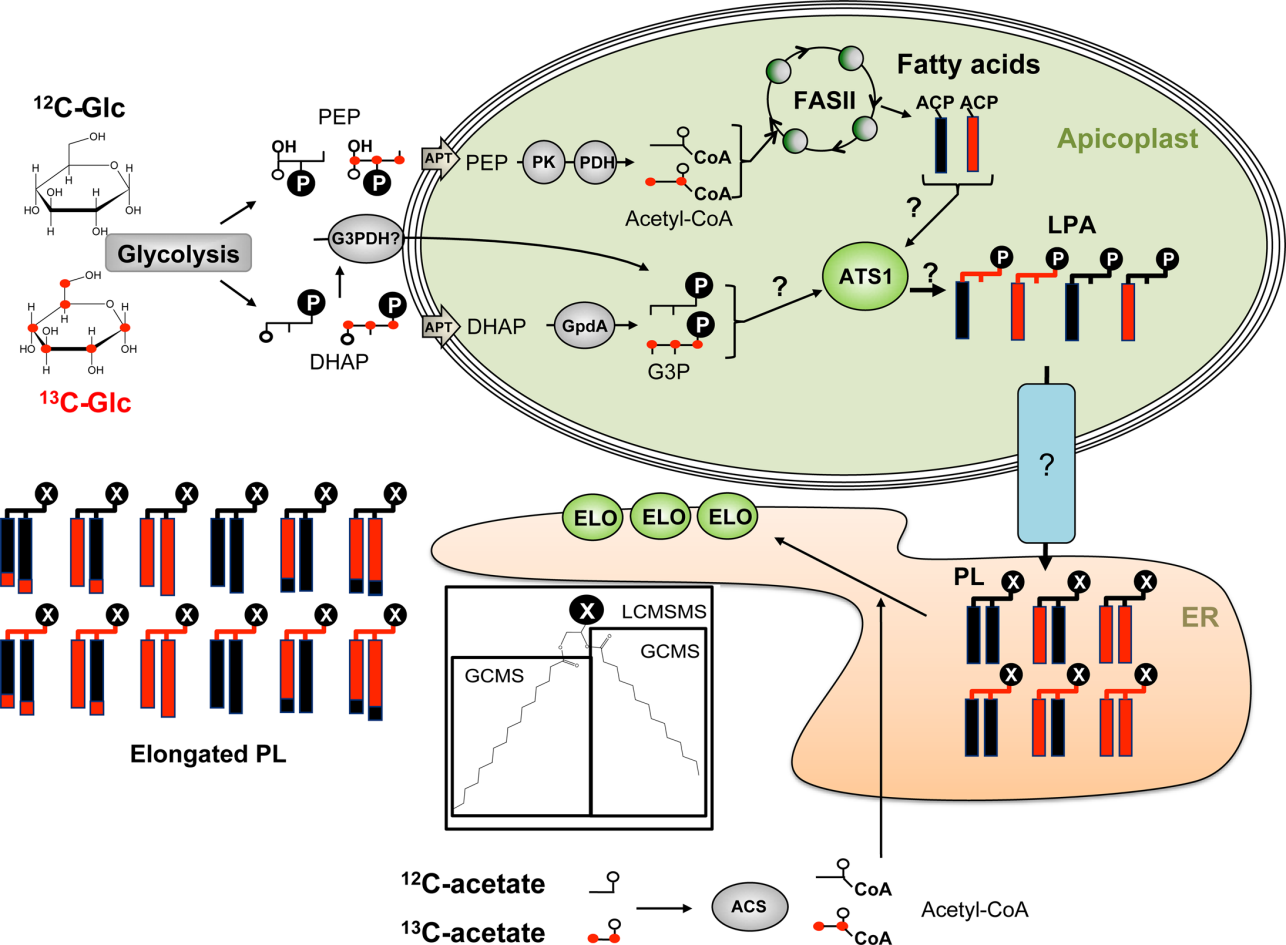
^13^C-carbon source labelling strategies to assess the role of FASII and *Tg*ATS1 in *T*. *gondii* membrane biogenesis. *TgATS1*-iKO parasites were grown in the presence of ^12^C-glucose or U-^13^C-glucose in the presence or absence of ATc. Glucose is metabolized via the parasite glycolytic pathway into dihydroxyacetone-phosphate (DHAP) and phosphoenolpyruvate (PEP). These precursors are imported into the apicoplast via the apicoplast phosphate transporter (APT). PEP can then be transformed into acetyl-CoA via the action of the apicoplast pyruvate kinase (PK) and the pyruvate dehydrogenase complex (PDH). Acetyl-CoA serves as a substrate for the FASII pathway to generate growing acyl chains on an acyl carrier protein (ACP) scaffold. Glycerol 3-phosphate (G3P) can be generated from imported DHAP by the apicoplast glycerol 3-phosphate dehydrogenase (Gpda), or potentially by an as yet unidentified cytosolic G3P dehydrogenase (G3PDH). Both labelled and/or unlabelled G3P and FA can be used as a substrate by the apicoplast *Tg*ATS1 (glycerol 3-phosphate acyltransferase) to form lysophosphatidic acid (LPA). These LPA species can potentially be exported from the apicoplast towards the ER to be assembled into phospholipids (PL) and/or have its FA elongated via the three ER elongases (ELO). In order to assess ER elongation, *TgATS1*-iKO parasites were grown in the presence or absence of ATc together with ^12^C-acetate or U-^13^C-acetate, which is metabolized to cytosolic acetyl-CoA by acetyl-CoA synthetase (ACS). Collectively, this strategy enabled the determination of (i) apicoplast FASII-generated FA products, (ii) products assembled via *Tg*ATS1, and (iii) PL species generated via the apicoplast. Total lipid extracts from *TgATS1*-iKO parasites grown in the described conditions were analyzed by two mass spectrometry techniques. GC-MS was used for the measurement of total FA profiles and LC-MS/MS was used to determine the relative amounts of ^13^C incorporation into individual PL molecular species. In the cartoon, unlabelled and labelled moieties are shown in black and red, respectively.

To determine the role of *Tg*ATS1 in lipid biosynthesis, ATc-treated and untreated *TgATS1*-iKO parasites were labelled with U-^13^C-glucose (i.e. glucose where all carbons are ^13^C) and the *de novo* synthesis of fatty acids assessed by mass isotopomer distribution (MID) analysis of total parasite fatty acids by gas chromatography-mass spectrometry (GC-MS) analysis. MID involves quantification of the molecular ions of individual unlabelled fatty acid methyl esters and corresponding isotopomers generated by incorporation of ^13^C-skeletons derived from glucose, after subtraction of natural abundance isotopomers. For example, the MID of the palmitoyl methyl ester (*m*/*z* 270) containing a backbone of 16 carbons involves quantification of all ions from *m*/*z* 270 (indicated as M0) to *m*/*z* 286 (indicated as M16). We have previously used this approach to distinguish the rate of FA synthesis via the plastid FASII and ER-based elongation pathways through sequential condensation of two-carbon units from ^13^C-malonyl-CoA onto the growing FA chain (Fig 4, [8, 12]).

**Fig 5:**
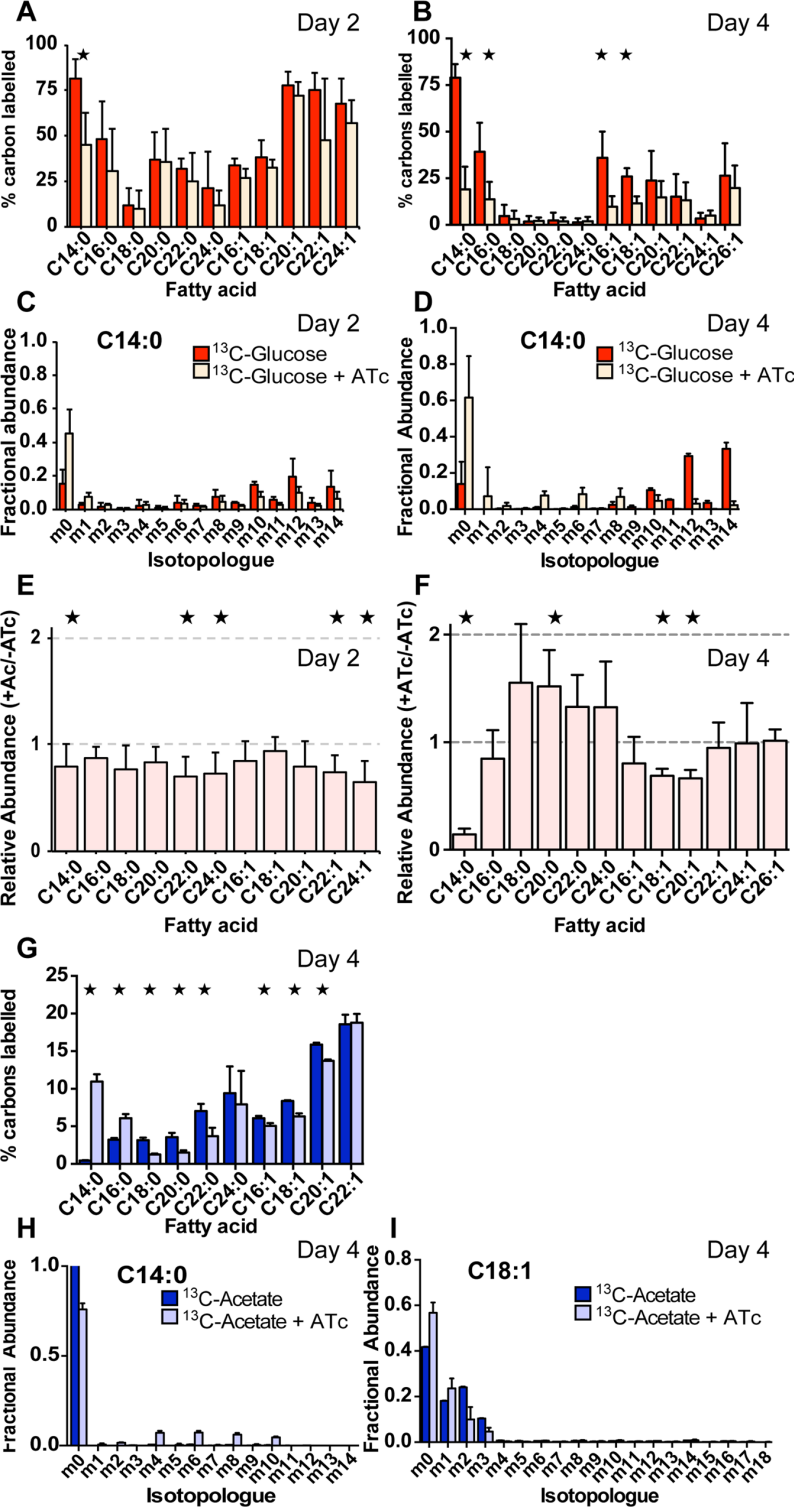
Analysis of FASII fatty acid biosynthesis and elongation in *TgATS1*-iKO parasites by metabolic labelling using stable isotope precursors. Tachyzoites of *TgATS1*-iKO parasites were labelled with U-^13^C-glucose for 2 or 4 days or U-^13^C- acetate for 4 days in the presence or absence of ATc. Lipids were extracted, derivatized to form fatty acid methyl esters (FAMEs), and analysed by GC-MS to determine ^13^C incorporation. **(A, B)** ^13^C incorporation into fatty acids from U-^13^C-glucose in the absence (dark colour) or presence (light colour) of ATc for 2 **(A)** and 4 days **(B)**. **(C, D)** Mass isotopologue distributions (MID) of FA (C14:0) from U-^13^C-glucose in the absence or presence of ATc for 2 **(C)** and 4 days **(D)** (colour scheme as above). The x-axis indicates the number of ^13^C atoms in each FAME, where ‘m0’ indicates the monoisotopic mass containing no ^13^C atoms, while ‘mX’ represents that mass with ‘X’ ^13^C atoms incorporated). MIDs for the all detected FAMEs using U-^13^C-glucose are shown in S5 Fig. **(E, F)** Changes in the overall abundance of FAMEs for *TgATS1*-iKO parasites grown in the absence or presence of ATc for 2 **(E)** and 4 days **(F)**. **(G)** ^13^C label incorporation rate into fatty acids from U-^13^C-acetate in the absence (dark colour) and presence (light colour) of ATc for 4 days. **(H, I)** MIDs for C14:0 **(H)** and C18:1 **(I)** labelled with U-^13^C-acetate in the absence (dark colour) and presence (pale colour) of ATc for 4 days. MIDs for the all detected FAMEs using U-^13^C-acetate are shown in S6 Fig. Error bars indicate standard deviation (n = 4 biological replicates). Stars represent significant (*p* < 0.05) differences as determined by t-test, corrected by the Holm-Sidak method.

Intracellular tachyzoites were labelled with U-^13^C-glucose for 1–4 days in the presence or absence of ATc, and total parasite FA was analysed by GC-MS. As expected, a wide range of FA were labelled in the absence of ATc (Fig 5A and 5B, S4A Fig) [12, 17, 53, 54]. Interestingly, incorporation was highest after 2 days and decreased by day 4, suggesting that parasites may switch from *de novo* synthesis to increased salvage of host fatty acids at later time points (Fig 5B). Inspection of MIDs revealed uniform labelling of some of the saturated FA, C14:0, C16:0, and unsaturated FA C16:1, confirming that labelling was due to (apicoplast located) *de novo* synthesis rather than (ER-located) elongation reactions (Fig 5C and 5D, S4B Fig and S5 Fig).

Labelling of parental cell lines with U-^13^C-glucose (or U-^13^C-acetate, see below) was unaffected by addition of ATc, while host cell lipids were not labelled in either condition [12]. Addition of ATc to the *TgATS1*-iKO did not significantly affect ^13^C-incorporation into C14:0, C16:0, C16:1, and C18:1 after 1 day (Day 1, S4A Fig), but resulted in a significant decrease of ^13^C-incorporation into C14:0 by day 2 (Day 2, Fig 5A), and C14:0, C16:0, C16:1, and C18:1 by day 4 (Fig 5B). Importantly, the fraction of C14:0 and C16:0 molecules that were labelled after 4 days of *TgATS1* repression incorporated ^13^C up to the full complement of 14 or 16 atoms, indicating that FASII was still active (Fig 5D, S5 Fig). Interestingly, while cellular abundance of all fatty acids was slightly (yet significantly for five of the 11 fatty acids) reduced after 2 days of *TgATS1* repression (Fig 5E), a different pattern was observed after 4 days (Fig 5F). Here, cellular abundance of C14:0 was greatly and significantly reduced, with smaller reductions observed for C16:0, C16:1, C18:1, and C20:1 (significantly for the latter two). Conversely, longer chain saturated fatty acids increased in abundance (significantly for C20:0) (Fig 5F). Taken together, this indicates that *Tg*ATS1 disruption results in reduced flux through the FASII pathway, which might be partly compensated by increased fatty acid recycling/remodelling after 4 days of *TgATS1* repression.

To investigate the effect of *Tg*ATS1 disruption on FA elongation, intracellular *T*. *gondii* tachyzoites were labelled with U-^13^C-acetate and harvested and analysed as above (see Fig 4 for labelling strategy schematic). U-^13^C-acetate, through conversion to ^13^C-acetyl-CoA and then ^13^C-malonyl-CoA, is efficiently used by the ER-localized elongase machinery, but not apicoplast FASII, providing a specific measure of elongation [12, 53, 54]. In the absence of ATc, high levels of ^13^C incorporation were observed in the longer chain (>C16) FA, with incorporation increasing with chain length (Fig 5G), as observed previously [53]. MID analysis suggested that ^13^C-acetate was incorporated onto unlabelled C14:0 or C16:0 to produce longer chain fatty acids (as observed by isotopologues sequentially increasing by 2 mass units for all FA 16 carbons and longer; Fig 5H and 5I, S6 Fig). After 4 days of *TgATS1* repression by ATc, ^13^C incorporation was significantly reduced for some FA (C16:1, C18:0, C18:1, C20:0, C20:1, and C22:0), although not all (Fig 5G). Interestingly, despite reduced abundance (Fig 5F), ^13^C incorporation significantly increased in C14:0 and C16:0. Taken together, these analyses suggest that loss of *Tg*ATS1 results in a selective defect in the synthesis of saturated long chain fatty acids in the apicoplast, but that this can be partially compensated for by increased elongation of fatty acids scavenged from the host cell.

### The apicoplast is responsible for biosynthesis of bulk phospholipid classes, most of which are generated via *Tg*ATS1

We hypothesised that *Tg*ATS1 may be required for incorporation of plastid-synthesised FA into LPA, an obligate intermediate of PA *de novo* synthesis (Fig 4). PA is the essential precursor for all *de novo*-synthesised PLs, which are bulk components of all cellular membranes, including those of the apicoplast. To assess the role of *Tg*ATS1 on PL biosynthesis, lipids from *TgATS1*-iKO parasites grown in the presence or absence of ATc were extracted and analyzed by liquid chromatography-mass spectrometry (LC-MS/MS) for the three major PL classes: PC, PI and PE. Precursor ion scanning was used for PC and PI (precursors of *m*/*z* 184 in positive mode and *m*/*z* 241 in negative mode, respectively) and neutral loss scanning was used for PE (neutral loss of 141 u in positive mode).

**Fig 6:**
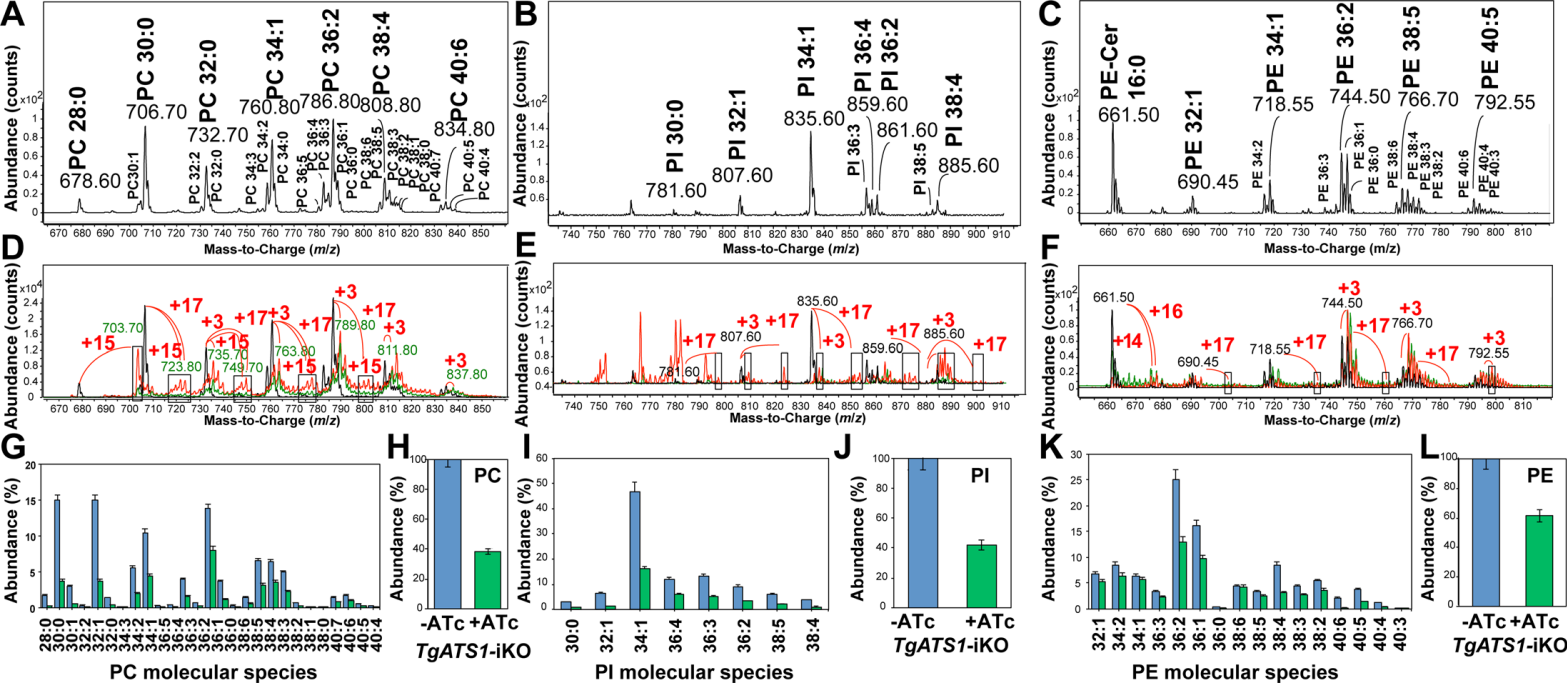
Metabolic labelling and LC-MS/MS analysis reveal that *Tg*ATS1 is responsible for the bulk assembly of PC, PI and PE. **(A, B, C)** Representative mass spectra of unlabelled PC **(A)**, PI **(B)**, and PE **(C)** species from *TgATS1*-iKO parasites grown in the absence of ATc. PC molecular species were obtained by *m*/*z* 184 precursor ion scan in positive mode. PI molecular species were obtained by *m*/*z* 241 precursor ion scan in negative mode. PE molecular species were obtained by 141 u neutral loss scan in positive mode. **(D, E, F)** Representative mass spectra of U-^13^C-glucose-labelled PC **(D)**, PI **(E)**, and PE **(F)** molecular species from *TgATS1*-iKO parasites grown in the absence (red line) or presence of ATc (green line) for 4 days. Labelled molecular species displayed typical mass shifts of +3, +15 and/or +17 corresponding to fully-labelled G3P, LPA(12:0) and LPA(14:0), respectively. Under ATc treatment, labelling was greatly reduced in the parasite, especially for +15 and +17 mass shifts. The black line represents a parental unlabelled control. **(G-L)** LC-MS/MS analysis of individual molecular species and relative abundance of PC **(G, H)**, PI **(I, J)** and PE **(K, L).** Parental parasite strain, shown in blue bars, and *TgATS1*-iKO parasites in the presence of ATc, shown in green bars (n = 3 with error bars representing standard deviation).

In the absence of ATc, LC-MS/MS revealed a PL repertoire in untreated (and parental) parasites (Fig 6A, 6B and 6C) similar to previous reports [16]. Our analyses detected 27 PC molecular species (Fig 6A), eight PI molecular species (Fig 6B), and 16 PE molecular species (Fig 6C), each class containing FA moieties of various lengths and degrees of saturation.

U-^13^C-glucose labelling can be used to assess *de novo* synthesis of LPA and PA by observation of label incorporation into FA (as explained above) and the glycerol 3-phosphate (G3P) backbone to which the FA moieties are attached (Fig 4 shows possible combinations of label incorporation into LPA and PA). U-^13^C-glucose incorporation into the glycerol and acyl moieties of each PL class was measured using PL-specific scanning, as above. Since choline and ethanolamine are not synthesised from glucose, and *T*. *gondii* lacks the Ino1 enzyme required for *de novo myo*-inositol synthesis, no PLs were observed with labelled head groups [31]. Substantial incorporation of ^13^C-atoms into G3P and FA was observed in PC, PI and PE (Fig 6D, 6E and 6F, respectively). The mass spectra of individual PL species contained +3, +15, or 17 isotopomers (Fig 6D, 6E and 6F), corresponding to labelling of (i) only the G3P backbone (i.e. where +3 corresponds to incorporation of 3 × ^13^C atoms into the glycerol of PL) or (ii) the G3P backbone and an acyl chain (i.e. where +15 corresponds to incorporation of 3 × ^13^C atoms into glycerol and 12 × ^13^C atoms into an acyl chain, while +17 corresponds to incorporation of 3 × ^13^C atoms into glycerol and 14 × ^13^C atoms into an acyl chain). MS/MS analyses confirmed that these PL molecules contained one labelled and one unlabelled acyl moiety (examples of MS/MS spectra for PI(36:4) are shown in S7 Fig). Therefore these PL molecules were assembled on LPA(C14:0) containing either 12 or 14 labelled carbons, correlating with the ^13^C incorporation observed in C14:0 (S5 Fig).

Due to the complexity of the spectra obtained in these analyses, it was difficult to confirm the full identities of all ^13^C-labelled lipids. We used a novel high-resolution chromatographic method to separate PL species of differing chain length, which allowed MS analysis of each molecular species (S8 Fig). This improved chromatography ensured that mass envelopes for each isotopologue series did not overlap, confirming that major PL species contained one or two labelled fatty acid moieties (an example of an MS/MS analysis for PC(30:0) is shown in S9 Fig). In all cases, the LPA moiety of the PL was labelled, providing direct evidence that apicoplast-synthesized FA can be assembled into LPA, thereby contributing to bulk PL composition.

Upon ATc repression of *TgATS1* expression for four days, the abundance of individual (Fig 6G) and total (Fig 6H) PC and individual (Fig 6I) and total (Fig 6J) PI was reduced by up to 60%. Inspection of mass spectra revealed that *TgATS1* repression led to a reduction in ^13^C label incorporation in both PC (Fig 6D) and PI (Fig 6E), as observed by the reduced levels of +15, +17 ^13^C-atoms in most PL isotopologues (compare red (-ATc) and green (+ATc) spectra), indicative of reduced LPA assembly and subsequent PL synthesis. These analyses suggest that *Tg*ATS1 assembles apicoplast-generated FA into the LPA precursor for PC and PI synthesis. Interestingly, while the abundance of individual (Fig 6K) and total (Fig 6L) PE was also reduced (by up to 40%) upon *TgATS1* repression, the effect on ^13^C incorporation was minimal (Fig 6F).

Neutral loss scanning for PE revealed the presence of an ion of *m*/*z* 661.53, which did not correspond to any predicted PE mass (Fig 6C), but was identified as PE-ceramide (d18:1/16:0) by MS/MS analysis (S10A Fig). The C16:0 moiety of this PE-ceramide contained up to 14 or even 16 ^13^C-atoms (S10B Fig), suggesting that ceramide is also synthesized using *de novo*-synthesized fatty acids from the apicoplast. The abundance of this lipid was not affected after 4 days of *TgATS1* repression (S10C Fig) and, although label incorporation was affected under these conditions, fully labelled FA was still present (S10B Fig), suggesting both that FASII was still active when *Tg*ATS1 is disrupted and that *Tg*ATS1 was not significantly responsible for PE-ceramide precursor production.

**Fig 7:**
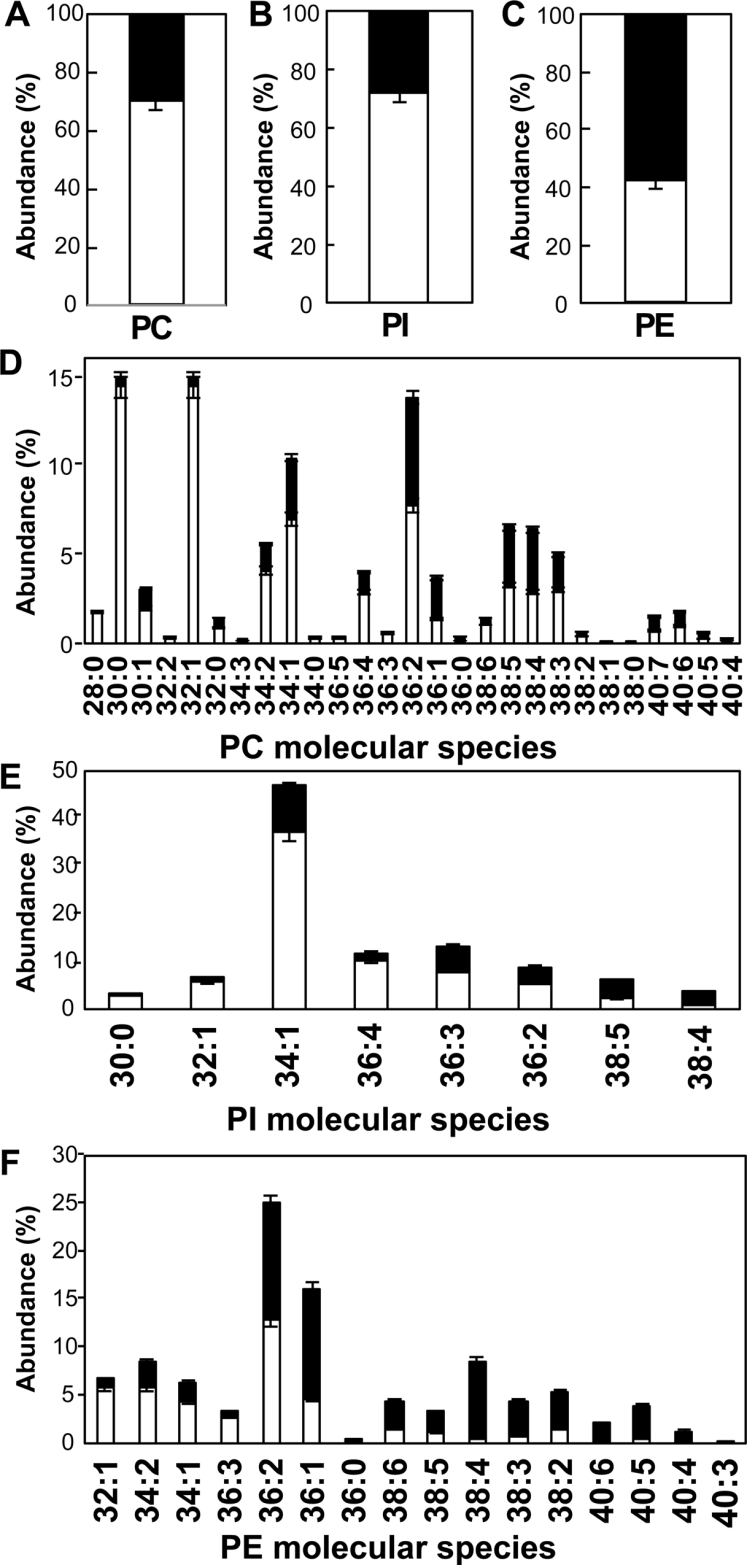
U-^13^C-glucose incorporation to fatty acids determines the apicoplast generates the FA moieties for production of most PC and PI molecular species. U-^13^C-glucose was incorporated into ATc-untreated parasites grown in glucose-free medium. LC-MS/MS analysis of labelled molecular species that have incorporated 4 or more ^13^C-atoms allowed quantification of apicoplast-generated species as shown in white, while all other species (mass shift +3) allowed quantification of non-apicoplast-generated species as shown in black. **(A, B, C)** Relative abundance for total PC **(A)**, PI **(B)** and PE **(C)**. **(D, E, F)** Relative abundance of individual molecular species for PC **(D)**, PI **(E)**, and PE **(F)**. The data shows that the apicoplast generates the FA moieties for production of ~70% PC, ~72% PI, and ~42% PE molecular species. Error bars indicate standard deviation, (n = 3 biological replicates).

To further investigate this apparent specificity in *Tg*ATS1 contribution to PL assembly, we quantified the relative amount of labelled (i.e. apicoplast-synthesised) and unlabelled (scavenged/remodelled) FA in PC, PI, and PE. Since the G3P backbone of all PLs may be generated by either a plastid or cytosolic G3P dehydrogenase (TGGT1_210260 or TGGT1_307570, respectively), we assumed that all species containing only 3 ^13^C-atoms were derived from a U-^13^C-G3P esterified to unlabelled (i.e. not apicoplast origin) FA, while all species that contained more than three ^13^C-atoms indicated a U-^13^C-G3P esterified to at least one labelled (i.e. apicoplast origin) FA. In untreated (and parental) parasites, the total amount of apicoplast-generated (i.e. labelled) FA was ~70%, 74% and 42% for PC, PI and PE, respectively (Fig 7A, 7B and 7C, respectively), where PLs with shorter FA moieties contained proportionally more apicoplast-generated FA than PLs with longer FA (Fig 7D (PC), 7E (PI) and 7F (PE)), correlating with our quantitative labelling analysis (Fig 6G, 6I and 6K, respectively). Specifically, PC species ≤38 and PI species ≤36 carbons were assembled from >50% apicoplast-derived FA (Fig 7D and 7E, respectively), while this was true for PE species of only ≤34 carbons in length (Fig 7F).

Precursor ion scans of *m*/*z* 241 for PI species also led to the identification of molecular species that were eventually identified as potential minor PE species. Fragmentation of these PE precursor ions indeed led to generation of *m*/*z* 241 in negative mode (S11 Fig). The analysis was also extended to the other PL species, namelyphosphatidylglycine (PG), phosphatidylserine (PS), and the newly discovered phosphatidylthreonine (PT) [55]. PG and PS were beneath the limit of quantification. The identification of two ions of *m*/*z* corresponding to published PT species (*m*/*z* 850.56, PT(40:5); *m*/*z* 878.59 PT(42:5)) was attempted. However, MS/MS fragmentation could not confirm the identity of these ions as being PT species

### *Tg*ATS1 is responsible for the synthesis of LPA (C14:0)

**Fig 8:**
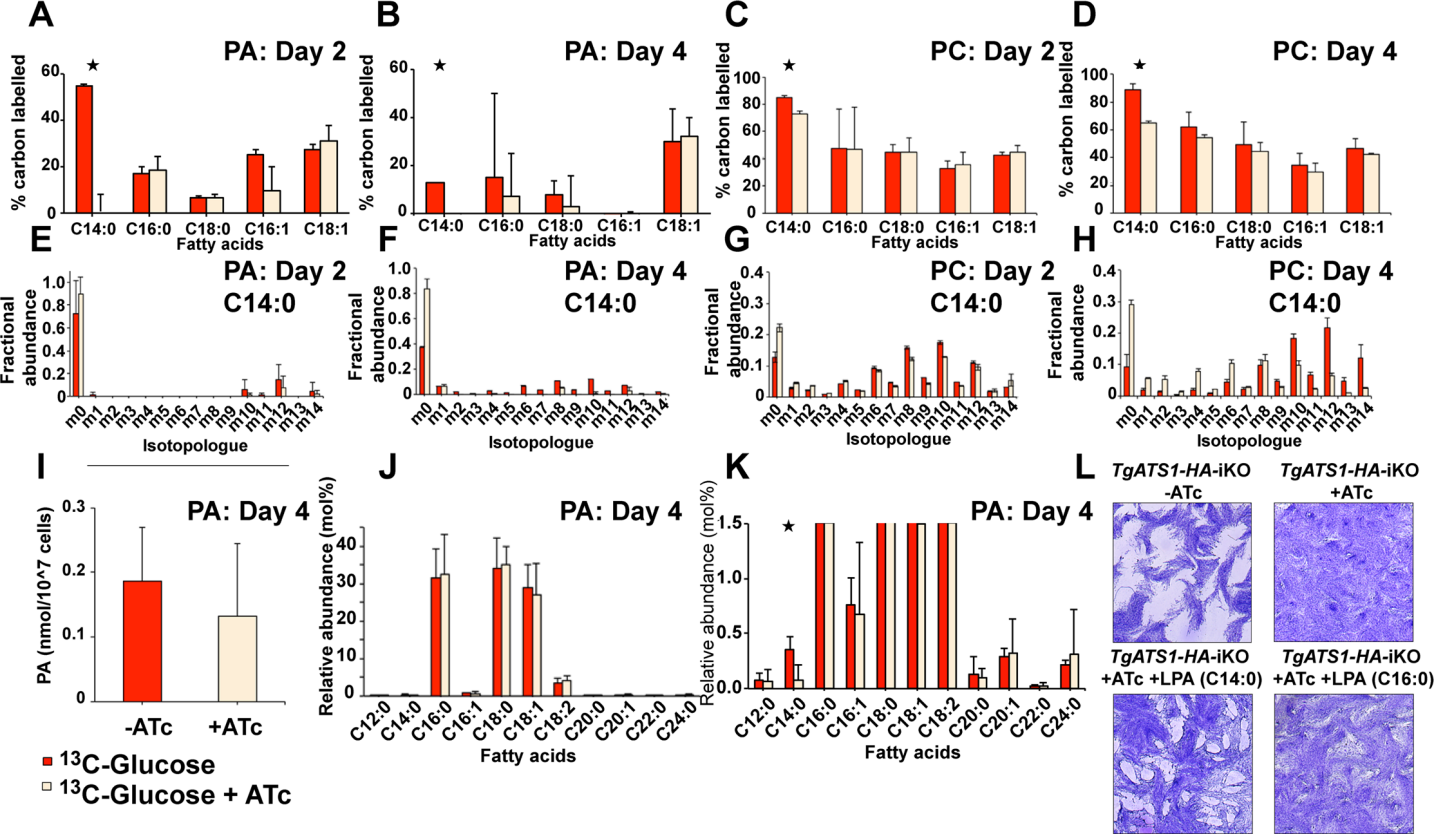
Analysis of PA and PC biosynthesis in *TgATS1*-iKO parasites using U-^13^C-glucose labelling. Tachyzoites of *TgATS1*-iKO parasites were labelled with U-^13^C-glucose in the presence (dark red) or absence (light red) of ATc for up to four days. Lipids were extracted, derivatized, and the resulting FAMEs were analysed by GC-MS to determine isotope incorporation. **(A, B)** Mean label incorporation from U-^13^C-glucose into PA in the absence or presence of ATc for 2 **(A)** and 4 days **(B). (C, D)** Mean label incorporation from U-^13^C-glucose into PC in the absence or presence of ATc for 2 **(C)** and 4 days **(D). (E-H)** Corresponding MIDs of C14:0 from panels A-D. ‘m0’ indicates the monoisotopic mass containing no ^13^C atoms, while ‘mX’ represents that mass with ‘X’ ^13^C atoms incorporated). **(I)** Quantification of PA after 4 days growth in the presence (dark red) or absence (light red) of ATc. **(J)** Abundance of each fatty acid species in PA, presented as a fraction (mol. %) of the total PA fatty acid pool (enlarged to show detail of low abundance FAMEs in **(K)**), colour scheme as above. For all analyses, error bars indicate standard deviation (n = 3 biological replicates). Stars indicate significant differences (*p* < 0.05) as determined by t-test, corrected by the Holm-Sidak method. **(L)** *TgATS1-HA*-iKO parasite plaque assay in the presence of LPA(C14:0) or LPA(C16:0). LPA(C14:0), but not LPA(C16:0), restored growth in the *TgATS1*-repressed (+ATc) parasites.

Since the putative product of *Tg*ATS1 is predicted to be LPA, the direct precursor to PA, label incorporation into LPA and PA was investigated. Despite our improved chromatographic resolution, we did not detect free LPA and PA by LC-MS. Instead, we purified PL species by two dimensional high performance thin layer chromatography (2D-HPTLC) and analysed individual species by GC-MS. While LPA remained undetectable, PA was observed (albeit in low amounts) and contained C14:0, C16:0, C16:1, C18:0, and C18:1 FA moieties (Fig 8A). After 2 days of *TgATS1* repression, ^13^C incorporation into PA(C14:0) was reduced significantly (Fig 8A), while incorporation into longer FAs (C16:0, C18:0) was reduced by day 4, albeit insignificantly (Fig 8B). Analysis of the PC spot (Fig 8C, day 2; 8D, day 4), confirmed the LC-MS results that ^13^C incorporation was significantly affected in PC species containing C14:0 FA. Importantly, the MID of PA(C14:0) in *TgATS1*-repressed parasites was very similar in pattern to, but of lower level than, the unrepressed control (Fig 8E, day 2; 8F, day 4), suggesting that (L)PA assembly is perturbed in these cells rather than FASII. The equivalent C14:0 MID in PC (Fig 8G, day 2; 8H, day 4), along with the relatively minor effects in label incorporation into PE and PE-ceramide (Fig 6F, S10B Fig), confirmed these results.

Although PA is downstream of the *Tg*ATS1 product LPA, we did not observe a significant reduction of total PA after 4 days of *TgATS1* repression (Fig 8I). However, quantification of the fatty acid repertoire of PA revealed a significant reduction of LPA(C14:0) in the presence of ATc (Fig 8J and 8K), suggesting that this lipid may be a major product of *Tg*ATS1. To investigate whether LPA(C14:0) is important for parasite survival, we investigated whether exogenous LPA could rescue cell growth in plaque assays. Infected fibroblasts were treated with/without ATc, in the presence or absence of LPA(C14:0) or LPA(C16:0) in the culture medium (Fig 8L). Strikingly, addition of LPA partially restored parasite growth, an effect that was only observed when using LPA(C14:0) (Fig 8L, lower left panel), and not LPA(C16:0) (Fig 8L, lower right panel). These data confirm that *Tg*ATS1 has a specific role in generating LPA(C14:0) using apicoplast-derived fatty acids and that this species is critic.al for other PL biosynthetic pathways.

## Discussion

Recent studies have shown that FA synthesized in the *T*. *gondii* apicoplast by the FASII enzyme complex can be trafficked to the parasite ER where they are further elongated and desaturated [12, 53, 54]. However, it remained unclear to what extent apicoplast-generated FA are incorporated into membrane phospholipids and why *de novo* FA biosynthesis cannot be bypassed by FA scavenging pathways.

Here we demonstrate that the apicoplast of *T*. *gondii* harbours a glycerol 3-phosphate acyltransferase, *Tg*ATS1, that is orthologous to the plant chloroplast ATS1 responsible for the initiation of the prokaryotic PA synthesis pathway. We show that *Tg*ATS1 is responsible for the recruitment of FASII-generated FA (most notably C14:0) into bulk synthesis of major phospholipid classes (PC, PE and PI) found throughout the parasite membranes and that this is critical for normal parasite growth and development, as also observed in *P*. *yoelii* liver stages [44]. A similar phenotype was observed in plants, although the loss of ATS1 function may be partially compensated by a second, ER-localised PA synthesis pathway of eukaryotic origin maintaining minimal plant development [56, 57]. Despite having this second pathway, a similar compensation is not observed in *T*. *gondii*. This may be because plant (and algal) ATS1 assembles FASII-generated FA only into plastid-specific lipids, including PG and galactolipids [56, 57]. In contrast, our data indicates that apicoplast lipid precursor synthesis has a much broader role in total lipid production in *T*. *gondii*, possibly as a result of having evolved to provide FA/lipids to the ER.

In algae and plants, FA and G3P can be assembled in the plastid via ATS1 to form LPA, which is the precursor for all other phospholipids. We show that *Tg*ATS1 is a typical, soluble algal/plant-like ATS based on sequence similarity and structural modelling. *Tg*ATS1 has a putative domain II containing all of the residues required for catalysis, including the catalytic pocket motif (NHX4D) arranged in a similar spatial arrangement to that of plant ATS1. Our results suggest that *Tg*ATS1 displays specificity regarding FA length (i.e. showing a preference for C14:0 over C16:0). Since FA specificity could have strong implications for membrane biogenesis during intracellular parasite development, FA selectivity should be addressed more closely in these parasites.

### *De novo* synthesis of fatty acids in the apicoplast

Using U-^13^C-glucose incorporation as a marker for FASII synthesis, we show here and in previous studies [54] that the apicoplast FASII machinery generates FA ranging from C12:0 to C16:0, and only a minor amount of C18:0. When *TgATS1* is repressed (by addition of ATc) for 4 days, we observed reduced ^13^C incorporation into most FA species (Fig 5B). Over the course of ATc treatment, we also observed severe disruption in apicoplast morphology and biogenesis, as well as changes in the IMC and most intracellular compartments (Figs 2C–2F and 3). We cannot distinguish between the possibilities that reduced ^13^C incorporation into FA is directly linked to the loss of *Tg*ATS1, or rather the loss of the apicoplast (and hence concomitant loss of FASII). The possibility that FA biosynthesis *per se* is not disrupted following repression of *Tg*ATS1 is suggested by the finding that ^13^C-incorporation into PE-ceramide was largely unaffected by *Tg*ATS1 repression, even after 4 days (S10B and S10C Fig). As ceramide synthesis is not dependent on *Tg*ATS*1* (S10B and S10C Fig) [58], this finding suggests that FASII is maintained in *Tg*ATS1-repressed cells, at least until the apicoplast is irrevocably damaged. Maintenance of FASII activity was also confirmed by (i) the late loss of the apicoplast after 5–6 days of *Tg*ATS1 repression (Fig 2E) and (ii) the detection of fully labelled FA moieties in PL after 4 days of *TgATS1* repression (Figs 5B, 6G–6L and 8B and 8D).

Using U-^13^C-acetate, we also observed ER-localized elongation of FA. This is a key difference from the plant chloroplast, where C16:0 and C18:0 typically constitute the major FASII end products (for a review, see [38]). Interestingly, when *TgATS1* is repressed, ^13^C incorporation from ^13^C-acetate is significantly increased in C14:0 (Fig 5G), most likely as an attempted compensatory mechanism responding to loss of C12:0 C14:0, C16:0 FA moieties. This suggests that the elongases could be used for *de novo* FA synthesis, or that ^13^C-acetate/^13^C-acetyl-CoA can enter the apicoplast and supply FASII when apicoplast integrity is compromised, although this is insufficient to support parasite growth. MID analysis of C14:0 (S6 Fig) suggests that an 8-carbon precursor (e.g. octanoate, C8:0) could be elongated under these conditions, perhaps reflecting increased scavenging of host lipids (such as lipoic acid) in an attempt to overcome reduced *de novo* PL assembly [46]. This increased scavenging may explain the low level of continued parasite growth observed after 8 days under *Tg*ATS1 repression (S3 Fig).

Most observed lipids/phospholipids are affected by the loss of ATS1 in *T*. *gondii*, most significantly in the decrease of C14:0 (Figs 5B and 8K) and those PLs derived from LPA containing C12:0 and C14:0 (Fig 6). Moreover, when labelling with U-^13^C-glucose, mature PL species with shorter FA (i.e. where the two acyl chains combined total 28 to 36 carbons) contained relatively more ^13^C-labelled FA than PLs with longer FA moieties (>36 carbons). These PL species were much more affected by *Tg*ATS1 loss (Fig 6). These results suggest that the parasite preferentially uses shorter, apicoplast-synthesised FA (C12-C18) for PL production. Longer chain (>C18) FA can also be used for PL synthesis, but are likely of non-plastid origin (e.g. via scavenging from the host, and/or remodelling of existing membrane lipids). Since only LPA(C14:0) could successfully rescue the loss of *Tg*ATS1 (Fig 8L), it is possible that LPA(C14:0) may act not only as the substrate for subsequent PL assembly but also in a signalling role to control overall lipid biosynthesis, although this remains to be confirmed.

### Function of the apicoplast in membrane biogenesis

Apicoplast FASII and ATS1 contribute to the synthesis of PL synthesis in the parasite, including the three PL classes, PC, PE and PI. This is a major departure from chloroplasts, the photosynthetic plastids of algae/plants, where the plastid-generated FA incorporated into LPA and PA via the PA synthesis pathway are solely used for the assembly of chloroplast lipids (i.e. galactolipids, sulfolipids and PG) [33]. Only under stressed conditions such as phosphate deprivation are chloroplast-derived glycerolipids transported to extra-plastidial membranes to compensate for loss of PL biosynthesis [42, 59]. Instead of generating galactolipids, apicomplexan parasites may have evolved to export LPA (and/or PA) from the apicoplast to the ER to generate PL for global membrane biogenesis. The machinery for export of LPA/PA is yet to be identified and may involve the contact sites observed between apicoplast and ER membranes in *T*. *gondii* [60]. Interestingly, plant chloroplasts possess a multimeric ATP-binding cassette (ABC) transporter that allows PL and possibly PA import into chloroplasts [61]. It is plausible that similar machinery may have evolved in Apicomplexa to export LPA/PA from the plastid, in a manner reminiscent of the change in function of the apicomplexan ER-associated protein degradation (ERAD) machinery allowing import, rather than export, of protein into the apicoplast [62].

The mechanism by which *Tg*ATS1-generated LPA is converted to PA and then PL remains unclear. The genome of *T*. *gondii* (and *Plasmodium* spp.) contains a hypothetical complete plant-like PA synthesis pathway that is predicted to be in the apicoplast, including ATS1 and a lysophosphatidic acid acyltransferase (LPAAT, or ATS2). Interestingly, immunofluorescence studies in *P*. *yoelii*, suggest that ATS2 (*Py*LPAAT) partially localises to the ER, despite the presence of a predicted N-terminal bipartite apicoplast-targeting sequence [44]. It will be of interest to determine whether *Tg*ATS2 resides in the ER, and hence whether it could convert LPA exported from the apicoplast to PA in the ER. Alternatively, ATS2 could be dually targeted to, or transported between, both organelles.

Continued synthesis of PLs may be required for transport of membrane vesicles to and from the apicoplast and the maintenance of apicoplast integrity. Consistent with this notion, we show that repression of *TgATS1* is associated with both the complete loss of the apicoplast organelles and/or the appearance of unusual vacuolar structures (Fig 3). In addition, internal compartment structure was highly affected upon the loss of *Tg*ATS1 (Figs 2F and 3), suggesting that *Tg*ATS1-generated PLs could be distributed throughout the cell to participate in global membrane biogenesis. Previous reports have hypothesised that apicoplast-derived FA may participate in apicoplast biogenesis in *T*. *gondii* [11] (and during liver stages of the rodent malaria parasites *P*. *berghei* and *P*. *yoelii* [14, 15, 44]). Our observations of defective apicoplast biogenesis in the *TgATS1* mutants support this idea, although we could not conclude whether this phenotype was due to a lack of PL for maintenance of apicoplast integrity or a general ‘loss of apicoplast/FASII’ phenotype. It would be interesting to assess the origin of *T*. *gondii* apicoplast membrane lipids, as has been done for *P*. *falciparum* [17].

We observed that while 70–74% of PC and PI fatty acids were of apicoplast-origin (Fig 7A and 7B, respectively), *TgATS1* repression resulted in the reduction of ~60% of PC and PI abundance (Fig 6H and 6J, respectively), suggesting that a considerable fraction (~10–15%) of PC and PI pools were also synthesised through this alternative, non-ATS1 route. The source of PA is not only from the ATS1-dependent apicoplast *de novo* pathway but also from PL recycling pathways, for example via DAG-kinase [63]. This likely explains why the loss of ATS1 did not significantly affect the total amount of PA (Fig 8J). Since PA is the central precursor for PL synthesis, the source(s) of PA should be proportionally reflected in all downstream PL. However, the loss of *Tg*ATS1 did not fully reflect this, since the incorporation of ^13^C label from U-^13^C-glucose into PC and PI was greatly affected by *Tg*ATS1 repression, while that of PE was less perturbed. This could be due to the difference in the substrate specificity of each synthetic pathway. For instance, elements/enzymes of the PC and PI biosynthetic pathways might have a preference for substrates with shorter acyl chains of apicoplast origin. Conversely, PE biosynthetic elements/enzymes might have a preference for longer acyl chains, likely derived from recycling pathways. Indeed, LC-MS/MS analyses revealed that PC and PI were present of shorter chain length (from PC(28:0) and PI(30:0)) than PE (from PE(32:1)) (Fig 7D–7F).

An alternative hypothesis could be due to the pathways downstream of ATS1 and ATS2. *De novo*-synthesized PA is usually converted to diacylglycerol (DAG) or cytidyldiphosphate-DAG (CDP-DAG) for subsequent PL synthesis. The *T*. *gondii* genome encodes two putative CDP-DAG synthetases (CDSs): TGGT1 281980 (*Tg*CDS1, a homolog to eukaryotic CDS), and TGGT1_263785 (*Tg*CDS2). *Tg*CDS1 (ER) and *Tg*CDS2 (apicoplast) have recently been localized and their disruption affects the synthesis of PI and PG, respectively [64]. This also supports the hypothesis that each CDS may have its own substrate specificity and, moreover, that this specificity may be related to the localization of these enzymes. The two putative *T*. *gondii* phosphatidic acid phosphatases (PAPs), generating DAG [63], have been localized in the cytosol or in the vicinity of the IMC of *T*. *gondii*, suggesting that the conversion of PA to DAG may occur outside the apicoplast, but any site-dependent substrate specificity is not yet known. It is not yet clear whether the specificity to the substrate determines enzyme localization or vice-versa. In addition, PE synthesis has been shown to rely on (i) ER-localized ethanolamine phosphotransferases (EPTs), (ii) conversion of PS to PE by a mitochondrial PS decarboxylase (*Tg*PDSmt), and (iii) a putative direct scavenging from the host cell [35]. These multiple sources and localizations of the PE biosynthetic pathways, especially those outwith apicoplast-ER interactions, may explain why PE is less affected by the loss of *Tg*ATS1 than PC and PI.

### Conclusion

**Fig 9:**
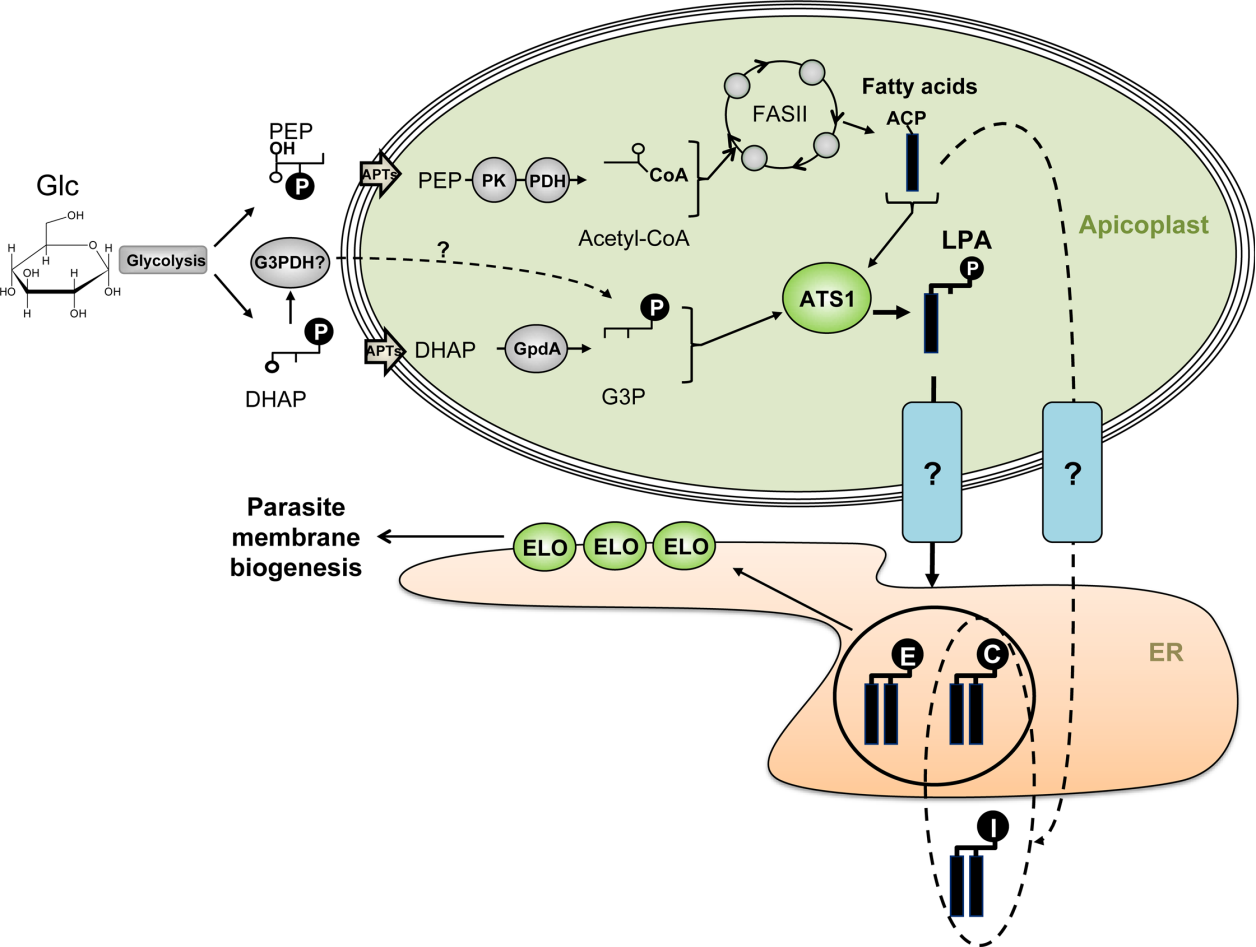
Proposed role of the apicoplast lipid assembly pathway. The glycolytic intermediates, dihydroxyacetone-phosphate (DHAP), and phosphoenolpyruvate (PEP) are imported into the apicoplast by the apicoplast phosphate transporter (APT) and converted to glycerol 3-phosphate (G3P) and acetyl-CoA, respectively. Acetyl-CoA is used by the FASII to generate, predominantly, C12 and C14 FA chains, which are transferred to G3P by *Tg*ATS1 to form lysophosphatidic acid (LPA). These LPA species are exported to the endoplasmic reticulum (ER) by an as yet unidentified transport system to generate bulk PC, PI and PE (shown as head groups with C, I and E, respectively). All PC, PI and PE species could then be elongated by elongase enzymes (ELO) before being exported to contribute to general parasite membrane biogenesis. *Note*: PI may also me assembled by PI synthase outside of the ER, most likely in the Golgi apparatus.

This study shows that apicoplast-generated FAs constitute an important source of precursors for bulk phospholipid biosynthesis during intracellular tachyzoite development. We show that apicoplast-synthesized FA are added to glycerol 3-phosphate by the enzyme *Tg*ATS1 to form LPA in the apicoplast. We propose that LPA is then converted to PA, and that LPA and/or PA are then trafficked to the ER, where they are ultimately converted to PC, PI, PE, and/or their precursors (DAG, CDP-DAG) (Fig 9). Mature PLs can then be trafficked to other organelles, including the apicoplast. Loss of *Tg*ATS1 is therefore associated with defects in PL synthesis and apicoplast biogenesis and function, including FA synthesis. *T*. *gondii*, and potentially other apicomplexan parasites, therefore appear to have redirected FASII/ATS1 pathways of FA/LPA synthesis from galactolipid biosynthesis (as occurs in plants and algal plastids) to PL synthesis for global membrane biogenesis. Although the precise location(s) of conversion of LPA to PA remains to be determined, it is clear that proteins involved in LPA synthesis and trafficking, including *Tg*ATS1, may be potential drug targets.

## Materials and Methods

### Sequence analysis, structure modelling, and phylogenetic analysis

A homology model of the *Tg*ATS1 structure was generated by threading the *Tg*ATS1 protein sequence (TGGT1_270910, www.toxodb.org) onto the crystal structure of *Cm*ATS1 (structure 1K30, www.rcsb.org, [47]) using Swissmodel (swissmodel.expasy.org, [65]) and Swiss PDB viewer. Robustness of the model was confirmed by a QMEAN4 score of 0.523 taking into account C-beta interaction energy, all atom pair-wise energy, solvation energy, and torsion angle energy [66]. Sequence alignments were generated using ClustalW using predicted protein sequences of *Tg*ATS1 (Toxodb.org, this work) *Pf*ATS1 (PF3D7_1318200, plasmodb.org), *Py*ATS1 PYYM_1420200, [44], plasmodb.org), *Cm*ATS1 [47, 67] and *At*ATS1 [68]. Secondary structure and putative residues involved in *Cm*ATS1 activity were retrieved from [47, 67]. Phylogenic tree of ATS1 related protein was created using following proteins: *Mycobacterium tuberculosis* CKU11951.1 *Mt*PlsB, *Escherichia coli* str. K-12 MG1655 AAC77011 *Ec*PlsB, *P*. *falciparum* PF3D7_1318200 putative *Pf*ATS1, *P*. *yoelii* PYYM_1420200 *Py*ApiG3PAT, *N*. *caninum* NCLIV_03587 putative *Nc*ATS1, *Hammondia*. *hammondi* HHA_270910 putative *Hh*ATS1, *T*. *gondii* TGME49_270910 *Tg*ATS1, *Emeria tenella* Houghton ETH_00020645 putative *Et*ATS1, *Chromera velia* CveI_26113 putative *Cv*ATS1, *Vitrella brassicaformis* Vbra_9602 putative *Vb*ATS1, *Phaelodactylum tricornutum* EEC47678 putative *Pt*ATS1, *C*. *moschata* BAB17755.1 *Cm*ATS1, *A*. *thaliana* OAP16056.1 *At*ATS1, *Thalassiosira pseudonana* XP_002292905.1 putative *Tp*ATS1, *Chlamydomonas reinhardtii* XP_001694977.1 putative *Cr*ATS1, *T*. *gondii* TGGT1_256980 putative *Tg*GPAT, *P*. *falciparum* PF3D7_1212500 *Pf*ERGPAT, *N*. *caninum* NCLIV_029980 putative *Nc*GPAT, P. berghei PBANKA_1428500 putative *Pb*GPAT, H. Hammondi HHA_256980 putative *Hh*GPAT, *Sarcocystis neurona* SRCN_2132 putative *Sn*GPAT, *E*. *tenella* ETH_00014360 putative *Et*GPAT, *Cryptosporidium parvum* CAD98671.1 putative *Cp*GPAT, *Theileria annulata* XP_954502.1 putative *Ta*GPAT, *V*. *brassicaformis* Vbra_5531 putative *Vb*GPAT, *C*. *velia* Cvel_20129 putative *Cv*GPAT, *T*. *pseudonana* CNC02160 putative *Tp*GPAT, *A*. *thaliana* AT1G01610 *At*GPAT, Homo sapiens NP_065969 *Hs*GPAT, *Paramecium tetraurelia* XP_001424966.1 putative *Pt*GPAT, *Tetrahymena thermophila* XP_001022288.2 putative *Tt*GPAT, *Saccharomyces cerevisiae* CAC85390.1 *Sc*GPAT. The analysis was performed on the Phylogeny.fr platform [69]. First, these protein sequences were aligned by ClustalW. After alignment, positions with gaps were removed from the alignment. Then, the phylogenetic tree was reconstructed using the maximum likelihood method implemented in the PhyML program (v3.0). The default substitution model was selected assuming an estimated proportion of invariant sites and 10 gamma-distributed rate categories to account for rate heterogeneity across sites. The gamma shape parameter was estimated directly from the data. Reliability for internal branching was assessed using the aLRT test (minimum of SH-like and Chi2-based parametrics). Graphical representation and edition of the phylogenetic tree were performed with TreeDyn (v198.3).

### *T*. *gondii* strains and cultures

*T*. *gondii* tachyzoites (RH-TATi and *TgATS1*-iKO)) were maintained in human foreskin fibroblasts (HFF) using Dulbecco’s Modified Eagle’s Medium (DMEM, Life Technologies) supplemented with 5% foetal bovine serum (Life Technologies), 2 mM glutamine and 25 μg/ml gentamicin at 37°C and 5% CO_2_.

### Construct design

The *TgATS1* open reading frame was PCR amplified using primers 5’-GATCTGATCAAAAATGCTTTTCTTTCTGGCTCGTCTCC and 5’-GATCTCTAGATTCGAAATGCGGAGTGATAAGTTTGTACAG, digested with *Bcl*I and *Xba*I, and ligated into the *Bgl*II and *Avr*II sites of vector pDt7s4H [51]. The resultant construct was transfected into TATi strain parasites and selected on pyrimethamine to produce the parental strain. This was subsequently cloned before characterisation. To disrupt the native *TgATS1* locus, we amplified the CAT cassette from the vector piCG [70] using primers 5’-TCACGAACCGAAACGAAAAACGTCCAATCCCTCTTCACAGACGGCCCAGGATACGACTCACTATAGGGCGAATTGG and 5’-GTCGAACGGATCCGGTTGGCTGAACACACCGTTGTCGTCTTGCCGGTCTCCCTCGACTACGGCTTCCATTGGCAAC. The resultant products were introduced into the cosmids TOXO218 and TOXOX36 using a recombineering approach, as described previously [52]. The resultant modified cosmids were transfected into the parental parasite strain expressing regulated *TgATS1*, selected on chloramphenicol, and cloned out. Clones were screened for successful disruption of the native *Tg*ATS1 gene using primers 5’-GCAGCAATAGTTCTTTTCAGG and 5’-AGGCGTCTTCGTGCGTATC, which will only give a band if the native *Tg*ATS1 gene is present. To further verify knockout of the native *Tg*ATS1 gene, Southern blotting was performed as described previously [71] using a probe amplified with the same primers as for the PCR screen.

### Antibodies and immunofluorescence assays

Primary anti-CPN60 (rabbit) antibodies [51] were used at a dilution of 1:3000, anti-IMC1 at 1:1000, anti-HA (Rat, Roche) at 1:500, and anti-ATRx1 (Mouse) at 1:1000. Secondary AlexaFluor 488- and 546-conjugated anti-rat and anti-rabbit antibodies (Life Technologies) were used at 1/10000 dilutions, respectively. Parasites were fixed in PBS containing 4% paraformaldehyde for 30 min on ice. Samples were permeabilized with 0.1% Triton X-100 in PBS for 10 min on ice before blocking in PBS containing 3% BSA and incubation with primary antibodies then secondary antibodies diluted in the blocking solution. Labelled parasites were stained with Hoechst (1:10,000, Life Technologies) for 20 min and then washed three times in PBS then H_2_O. Coverslips were mounted onto slides prior to observation using a Zeiss epifluorescent microscope.

### Electron microscopy

Intracellular tachyzoites were fixed in PBS containing 2.5% glutaraldehyde and 0.5% tannic acid (pH 7.2; Polysciences) for 15 min, followed by three washes in PBS. Samples were post-fixed in osmium tetroxide (OsO_4_) in PBS for 2.5 min followed by three washes in PBS and water. Samples were stained overnight in 1% uranyl acetate, washed three times in water and dehydrated in a graded series of ethanol. Samples were embedded in LR white resin (London Resin) and ultrathin sections were observed on a Phillips Bio Twin (120 kV) electron microscope.

### Phenotypic analyses

*Plaque assays*: HFF monolayers were infected with 500 parasites and allowed to develop under normal culture conditions for 10 days before staining with Crystal Violet (Sigma) and cell growth assessment by light microscopy for the presence of intact HFF.

*Apicoplast quantification assay*: *TgATS1*-iKO-FNR-RFP parasites were collected promptly after egress and inoculated onto new HFF monolayers and cultured for 0 to 6 days in the presence or absence of ATc. At each time point cultures were fixed with paraformaldehyde and stained with anti-IMC1 and anti-HA. The numbers of parasites per vacuole were counted for more than 100 vacuoles for each condition at each point time. Apicoplast loss was determined by observing the fluorescent FNR-RFP protein as an apicoplast marker.

*Intracellular growth assays*: We introduced cytosolic tdTomato into *TgATS1*-iKO parasites and performed fluorescence growth assays as described previously [71].

### Lipid extraction and analysis of *T*. *gondii* tachyzoites

Lipid extraction and analysis of tachyzoites was performed as previously described [12]. Intracellular tachyzoites (4 × 10^8^ cell equivalents) were extracted in chloroform/methanol/water (1:3:1, v/v/v containing 50 nmol laurate (C12:0) as internal standard) for 1 h at 4°C, with periodic sonication. For MS analysis, polar and apolar metabolites were separated by phase partitioning. Total fatty acid analysis of the lipophilic fraction was analysed by GC-MS (fatty acids) and LC-MS (phospholipids). In all cases, lipids were extracted from the same cell number and experiments were repeated using each independent mutant line as a biological replicate.

### Stable isotope labelling of *T*. *gondii* fatty acids and phospholipids

Stable isotope labelling using U-^13^C-glucose or U-^13^C-acetate (Cambridge Isotope Laboratories, USA), lipid extraction, and GC-MS analysis was performed as previously described [12, 53]. Briefly, freshly infected HFF were incubated in the presence or absence of ATc (0.5 μM, Sigma-Aldrich) in either glucose-free medium supplemented with 8 mM U-^13^C- glucose or low-glucose DMEM, supplemented with 8 mM U-^13^C- acetate. In *TgATS1* repression experiments, the ^13^C-carbon source was added simultaneously with ATc. Other supplements (glutamine, sodium bicarbonate, and foetal bovine serum) were added according to normal culture conditions. Parasites were harvested at indicated time points and metabolites extracted and partitioned as above. An aliquot of the lipid extract was derivatised on-line using MethPrep II (Alltech) and the resulting fatty acid methyl esters were analysed by GC-MS as previously described [12, 53]. All fatty acids were identified by comparison of retention time and mass spectra with authentic chemical standards and label incorporation was calculated as the percent of the metabolite pool containing one or more ^13^C atoms after correction for natural abundance and the amount of ^13^C-carbon source in the culture medium (as determined by GC-MS analysis). In these experiments, U-^13^C-glucose or U-^13^C-acetate were added at the same time as ATc, and so the label incorporation observed in +ATc samples may be from the initial period before *Tg*ATS1 was fully absent.

### Liquid chromatography-mass spectrometry analysis

Total lipids were extracted and partitioned as above. These extracts were suspended in 100 μL 1-butanol/10 mM ammonium formate in methanol (1:1, v/v) and 0.5 μL aliquots were analysed using the following LC-MS method. An Agilent 1290 series liquid chromatography (LC) system (maximum pressure 1200 bar) comprising a vacuum degasser, binary pump, column oven and temperature controlled autosampler was interfaced with a Jetstream electrospray ionization triple Quadrupole (Agilent 6490 QQQ) or Quadrupole Time-of-Flight (Agilent 6550 QTOF) mass spectrometer. The LC parameters were as follows: column: 2.1 × 100 mm, 1.8 μm C18 Zorbax Elipse plus (Agilent); column temperature: 60°C; rate 0.6 mL/min. Gradient elution was from 45% mobile phase B to 100% B over 20 min, followed by 5 min at 100% B and a 3 min re-equilibration to 45% B. Mobile phase A: 10 mM ammonium formate in water; mobile phase B: water:acetonitrile:isopropanol, 5:20:75 (v/v/v) with 10 mM ammonium formate. The ESI source settings were: gas temperature: 250°C; gas flow rate: 20 L/min; nebulizer pressure: 45 psi; sheath gas temp: 350°C; sheath gas flow: 11 L/min; capillary voltage: 3000 V. In-spectrum calibration of the QTOF data was performed using reference ions of 121.0508 *m/z* and 922.0097 *m/z* which were supplied through the second ESI needle. A 10,000-count threshold was set for untargeted MS/MS experiments. All solvents were LC-MS grade (Burdick and Jackson) and 18.2 MΩ deionized water used.

The following scans were used for the three key lipid classes on the QQQ-MS: PC positive ionisation precursor scan of *m*/*z* 184, PE (and PE-Cer) positive ion neutral loss scan of 141 u, and PI negative ionisation precursor ion scan of *m*/*z* 241. These correlate to the relevant polar head groups. Data were analysed using Mass Hunter Qualitative (QTOF data) and Quantitative (QQQ data) software (Agilent).

### PA quantification

Total lipid spiked with 25 nmol C13:0 fatty acid was extracted from U-^13^C-glucose labelled parasites prepared as above using chloroform:methanol, 1:2 (v/v) and chloroform:methanol, 2:1 (v/v) in the presence of 0.1 M HCl. Pooled organic phase was subjected to biphasic separation by adding 0.1 M HCl. The organic phase was dried under N_2_ gas and dissolved in 1-butanol. Total lipid was then separated by 2D-HPTLC with 1 μg PA(C17:0/C17:0) (Avanti Polar lipids) using chloroform/methanol/28% NH_4_OH, 60:35:8 (v/v) as the 1^st^ dimension solvent system and chloroform/acetone/methanol/acetic acid/water, 50:20:10:13:5 (v/v) as the 2^nd^ dimension solvent system [72]. The spot corresponding to PA was identified according to the migration of authentic PA standard, and subsequently extracted for GC-MS analysis (Agilent 5977A-7890B) after methanolysis using 0.5 M HCl in methanol incubated at 100°C for 1 h. Fatty acid methyl esters were identified by their mass spectrum and retention time compared to authentic standards. PA content was normalized according to the parasite cell number and internal standard.

## Supporting Information

S1 Fig Related to Fig 1: A three-dimensional structure of *Tg*ATS1 was generated using *Cm*ATS1 as a model [47]. **(A)** The overall structure as observed in the ribbon representation of *Cm*ATS1α-carbons. Surface accessibility **(B)** and residues involved in substrate binding (G3P and acyl-ACP) and the catalytic motif NHX4D **(C)** of *Cm*ATS1. The structure **(D)**, surface accessibility **(E)**, and residues and motifs **(F)** are conserved and highly similar in *Tg*ATS1, forming similar grooves and pockets to those found in *Cm*ATS1 **(G)** Phylogeny of *T*. *gondii* ATS1. Maximum likelihood phylogenies for the glycerol acyl transferases of 32 species. Branch support values are indicated in different colors (0–25, purple; 25–50, green; 50–75, orange; 75–100, red). The distance between each node is indicated in the Fig.
(TIF)

S2 Fig Related to Fig 2: Deletion and isolation of a conditional *Tg*ATS1 mutant by promoter replacement in the TATi transactivator line. Schematic representation of the two-step genome modification used to obtain a conditional *Tg*ATS1 mutant. **(A)** The *Tg*ATS1 sequence was fused (i) to a HA-tag coding sequence at its 3’-terminus and (ii) to the tetracycline inducible promoter sequence (Pi) at its 5’-terminus (i*Tg*ATS1). The construct was transfected and randomly inserted into the TATi line genome, prior to endogenous gene (e*Tg*ATS1) replacement by a Chloramphenicol Acyltransferase (CAT) resistance cassette [73] via double homologous recombination using a specific *CAT ATS1 KO* cosmid [74]. Probes and restriction sites used for Southern blot are indicated by arrowheads and restriction enzymes (RE) names, respectively. **(B)** Schematic representation of homologous recombination between the *CAT KO* cosmid and *TgATS1* locus. Probe and restriction sites used for Southern blot are indicated by arrowheads and RE names. **(C)** PCR confirms loss of endogenous copy of e*TgATS1* (arrows showing the positive clones). **(D)** Southern blot analysis of the i*TgATS1*/Δ*TgATS1* clone and its parental i*TgATS1*/e*Tg*A*TS1* line confirming e*TgATS1* disruption presence of i*TgATS1*.
(TIF)

S3 Fig Related to Fig 2: Real time fluorescence assay of *TgATS1*-iKO intracellular growth. Parasite growth rate was analysed over the course of 8 days by quantifying the fluorescence of tdTomato [71] expressed in the cytosol of *TgATS1*-iKO parasites in the absence (blue rectangles, control) or the presence of ATc. *TgATS1*-iKO were grown in the presence of ATc from day 0 to day 8 (red squares) or pre-treated with ATc for 3 days prior to the 8 day ATc treatment (green triangles). In the absence of ATc, *TgATS1*-iKO grew normally as observed by fluorescence levels but the presence of ATc substantially affected the amount of fluorescence, with this effects being strongest with 3 days of pre-treatment.
(TIF)

S4 Fig Related to Fig 5: Analysis of FASII fatty acid biosynthesis in *TgATS1*-iKO parasites by 1 day U-^13^C-glucose metabolic labelling using stable isotope precursors. Tachyzoites of *T*. *gondii* conditional mutants for ATS1 were labelled with U-^13^C-glucose for 1 day in the presence or absence of ATc. Lipids were extracted, derivatized, and the resulting FAMEs were analysed by GC-MS to determine ^13^C incorporation. **(A)** The mean label incorporation from U-^13^C-glucose into fatty acids is shown for parasites grown in the absence (dark red) and presence (light red) of ATc. **(B)** The MIDs for C14:0 labelled with U-^13^C-glucose in the presence (red) and absence (pale red) of ATc. The x-axis indicates the number of ^13^C atoms in each FAMEs (‘m0’ indicates the monoisotopic mass containing no ^13^C atoms, while ‘mX’ represents that mass with ‘X’ ^13^C atoms incorporated). Nomenclature Cx:y is shown where x is the number of carbons and y is the number of double bonds in the fatty acid chain. Error bars indicate standard deviation, where n = 2 biological replicates. Data shown has been background-subtracted for natural isotope abundance.
(TIF)

S5 Fig Related to Fig 5: Analysis of FASII fatty acid biosynthesis and elongation in *TgATS1*-iKO parasites by metabolic labelling using U-^13^C-glucose. Tachyzoites of *T*. *gondii* conditional mutants for *TgATS1* were labelled with U-^13^C-glucose in the presence or absence of ATc. Lipids were extracted, derivatized, and the resulting fatty acid methyl esters (FAMEs) were analysed by GC-MS to determine isotope incorporation. MIDs for all detected FAMEs labelled with U-^13^C-glucose in the absence and presence of ATc are shown in red and pale red, respectively. The x-axis indicates the number of ^13^C atoms in each FAMEs (‘m0’ indicates the monoisotopic mass containing no ^13^C atoms, while ‘mX’ represents that mass with ‘X’ ^13^C atoms incorporated). Nomenclature Cx:y is shown where x is the number of carbons and y is the number of double bonds in the fatty acid chain. Error bars indicate standard deviation, where n = 4 biological replicates. Data shown has been background-subtracted for natural isotope abundance.
(TIF)

S6 Fig Related to Fig 5: Analysis of elongation (and fatty acid biosynthesis) in *TgATS1*-iKO parasites by metabolic labelling using U-^13^C-acetate. Tachyzoites of *TgATS1*-iKO parasites were labelled with ^13^C-U-acetate in the presence or absence of ATc. Lipids were extracted, derivatized, and the resulting FAMEs were analysed by GC-MS to determine isotope incorporation. MIDs for all detected FAMEs labelled with U-^13^C-acetate in the absence and presence of ATc are shown in purple and pale purple, respectively. MIDs suggested that saturated/monounsaturated FAs incorporated ^13^C-atoms onto C16:0 or C14:0 in a units of two (i.e. C18 incorporated 2 or 4, C20 incorporated 4 or 6, C22 incorporated 6 or 8, and C24 incorporated 8 or 10 ^13^C-atoms onto C16:0 or C14:0, respectively). The x-axis indicates the number of ^13^C atoms in each FAME (‘m0’ indicates the monoisotopic mass containing no ^13^C atoms, while ‘mX’ represents that mass with ‘X’ ^13^C atoms incorporated). Nomenclature Cx:y is shown where x is the number of carbons and y is the number of double bonds in the fatty acid chain. Error bars indicate standard deviation, where n = 4 biological replicates. Data shown has been background-subtracted for natural isotope abundance.
(TIF)

S7 Fig Related to Fig 6: Representative MS/MS fragmentation confirms the presence of a ^13^C-labelled LPA backbone in PI. **(A)** Negative ion mode MS/MS fragmentation of unlabelled PI(36:4), *m*/*z* 857.51, extracted from *TgATS1-HA*-iKO cells grown in the absence of ATc. Characteristic fragment ions were detected as follows: LPA(20:4), *m*/*z* 439.22; LPA(16:0), *m*/*z* 391.22; FA(C20:4), *m*/*z* 303.23; FA(C16:0), *m*/*z* 255.28; inositol phosphate, *m*/*z* 241.01; glycerol 3-phosphate (G3P), *m*/*z* 152.99. **(B)** Equivalent MS/MS fragmentation of ^13^C-labelled PI(36:4), *m*/*z* 876.5711. Fragment ions corresponding to labelled and unlabelled moieties were observed as follows: LPA(20:4) containing labelled G3P, *m*/*z* 442.22; LPA(16:0) containing labelled G3P and labelled C16:0, *m*/*z* 410.29; labelled C16:0, *m*/*z* 271.28; G3P M+3, *m*/*z* 156.00.
(TIF)

S8 Fig Related to Fig 6: High-resolution chromatography allows intra-class separation of PL species. Chromatograms of PC, PE and PI extracted from *TgATS1-HA*-iKO parasites grown for four days in unlabelled conditions (black line) or ^13^C-glucose-labelled conditions in the absence (red line) or presence (green line) of ATc. **(A)** Total ion chromatogram from *m*/*z* 184 precursor ion scan in positive mode (for PC). **(B)** Total ion chromatogram from *m*/*z* 241 precursor ion scan in negative mode (for PI). **(C)** Total ion chromatogram from 141 u neutral loss scan in positive mode (for PE).
(TIF)

S9 Fig Related to Fig 6: Representative MS/MS fragmentation confirms the presence of a ^13^C-labelled moieties in PC. **(A)** Chromatogram of PC species determined by *m*/*z* 184 precursor ion scanning in positive mode for parental strain (black line) and *TgATS1*-iKO grown with U-^13^C-glucose in the absence of ATc (red line). PC(30:0) elutes at 13.27 min (blue frame). **(B, C)** Corresponding mass spectra and extracted ion chromatograms of PC eluting at 13.27 min. **(B)** A single peak of *m*/*z* 706.54 corresponding to PC(30:0) elutes in the parental strain (black line), whereas a series of peaks ranging from m/*z* 706.54 to 739.6 elute in the U-^13^C-glucose-labelled *TgATS1*-iKO (red line). **(C)** Extracted ion chromatogram of marked ion peaks from **(B)** all overlap precisely at 13.27 min (arrowheads) and they have similar peak shapes, indicating that they are isotopologues of the same molecule. **(D)** Putative PC(30:0) isotopologue structures of the multiple 13.27 min ion peaks from labelled *TgATS1*-iKO as follows: *m*/*z* 706.54, unlabelled PC(30:0); *m*/*z* 709.56, PC(30:0) containing labelled G3P; *m*/*z* 721.59, PC(30:0) containing labelled G3P and C14:0 labelled with 12 ^13^C atoms; *m*/*z* 723.59, PC(30:0) containing fully-labelled LPA(14:0); and *m*/*z* 737.64 PC(30:0) containing labelled G3P, fully-labelled C14:0 and C16:0 labelled with 14 ^13^C atoms. The *m*/*z* 737.64 ion may also represent PC(30:0) containing labelled G3P, fully-labelled C16:0 and C14:0 labelled with 12 ^13^C atoms. **(E, F)** Representative MS/MS fragmentation of annotated PC molecules. This confirmed that all ^13^C-labelled molecules observed in the spectrum were PC(30:0) by detection of typical *m*/*z* 184 phosphocholine polar head.
(TIF)

S10 Fig **Related to Fig 6: LC-MS/MS structure confirmation of PE-Ceramide(d18:1/16:0) (A)** Fragmentation of *m*/*z* 661.52 confirmed the assignment of the peak as PE-Cer(d18:1/16:0) by detection of the characteristic ions of ceramide and ethanolamine head group in positive mode: *m*/*z* 142 corresponds to ethanol amine phosphate; *m*/*z* 520.50 to sphingosine (d18:1/16:0) and *m*/*z* 264.2 to ceramide. **(B)** MIDs of PE-Cer(d18:1/16:0) extracted from *TgATS1*-iKO parasites grown in unlabelled conditions (black bars) or labelled with U-^13^C-glucose for 4 days in the absence (white bars) or presence of ATc (grey bars). ‘m0’ indicates the monoisotopic mass containing no ^13^C atoms, while ‘mX’ represents that mass with ‘X’ ^13^C atoms incorporated. **(C)** Relative abundances of apicoplast-generated FA moieties (i.e. those containing 4 or more ^13^C atoms) in PE-Cer(d18:1/16:0) extracted from *TgATS1*-iKO parasites grown in the presence and absence of ATc shows that the majority of FA is generated in the apicoplast and was not greatly affected by the disruption of *Tg*ATS1.
(TIF)

S11 Fig Related to Fig 6: MS/MS fragmentation of undetermined species detected by precursor ion scan for *m*/*z* 241 in negative mode suggests precursor ions corresponding to PE. **(A)** Negative ion mode MS/MS fragmentation of *m*/*z* 736.4879 eluting at 13.94 min reveals the presence of ions corresponding to FA(C22:5), *m*/*z* 329.24; FA(C20:4), *m*/*z* 303.23; FA(C16:1), *m*/*z* 253.21; and FA(C14:0), *m*/*z* 227.19. **(B)** Detail from panel A. Presence of a *m*/*z* 140.01 ion suggests that this ion could be a mix of PE(16:1/20:4) and PE(14:0/22:5). **(C)** Negative ion mode MS/MS fragmentation of *m*/*z* 790.5364 eluting at 15.16 min revealed the presence of ions corresponding to FA(C22:5), *m*/*z* 329.24; FA(C18:1), *m*/*z* 281.24; and, potentially, ethanolamine-phosphate, *m*/*z* 140.01. This suggests that the mass *m*/*z* 790.5364 corresponds to PE(18:1/22:5).
(TIF)
