# Supplementary figures and images for: Apicoplast-Localized Lysophosphatidic Acid Precursor Assembly Is Required for Bulk Phospholipid Synthesis in Toxoplasma gondii and Relies on an Algal/Plant-Like Glycerol 3-Phosphate Acyltransferase
Source: PLoS Pathog. 2016 Aug 4;12(8):e1005765. doi: 10.1371/journal.ppat.1005765 (PMC4973916; doi:10.1371/journal.ppat.1005765)

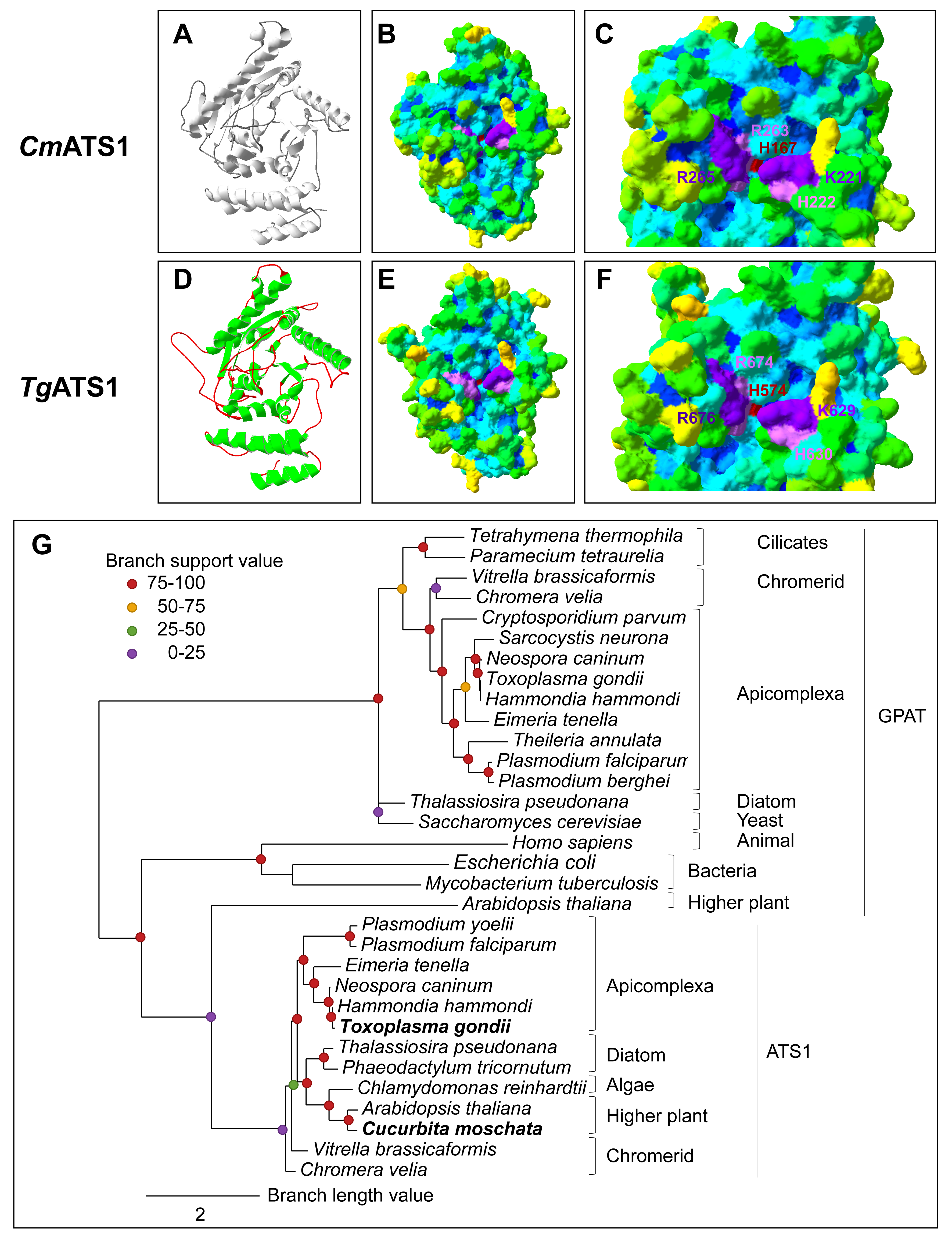

Supplement: S1 Fig — (A) The overall structure as observed in the ribbon representation of CmATS1α-carbons. Surface accessibility (B) and residues involved in substrate binding (G3P and acyl-ACP) and the catalytic motif NHX4D (C) of CmATS1. The structure (D), surface accessibility (E), and residues and motifs (F) are conserved and highly similar in TgATS1, forming similar grooves and pockets to those found in CmATS1 (G) Phylogeny of T. gondii ATS1. Maximum likelihood phylogenies for the glycerol acyl transferases of 32 species. Branch support values are indicated in different colors (0–25, purple; 25–50, green; 50–75, orange; 75–100, red). The distance between each node is indicated in the Fig. (TIF) [file ppat.1005765.s001.tif]

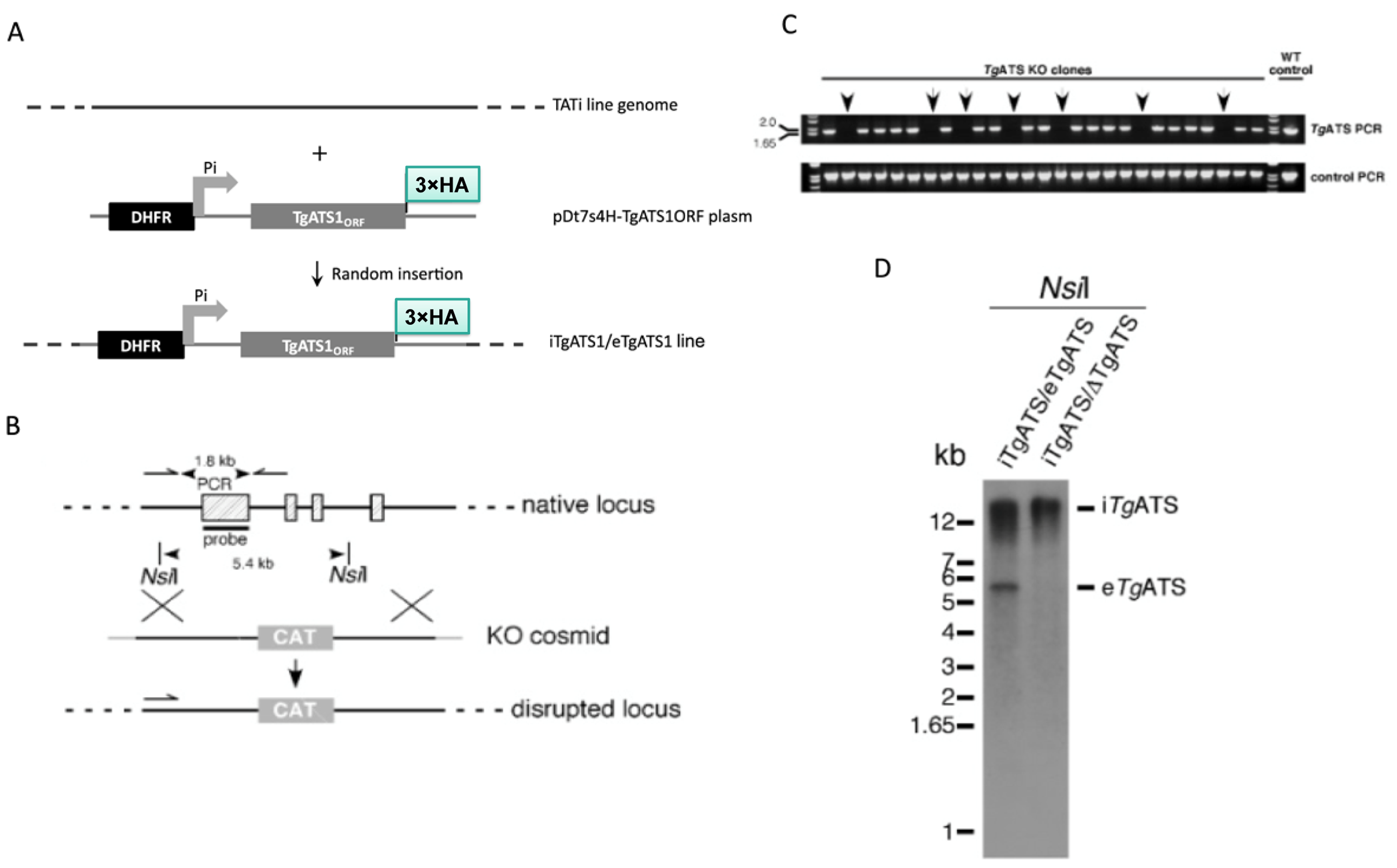

Supplement: S2 Fig — Schematic representation of the two-step genome modification used to obtain a conditional TgATS1 mutant. (A) The TgATS1 sequence was fused (i) to a HA-tag coding sequence at its 3’-terminus and (ii) to the tetracycline inducible promoter sequence (Pi) at its 5’-terminus (iTgATS1). The construct was transfected and randomly inserted into the TATi line genome, prior to endogenous gene (eTgATS1) replacement by a Chloramphenicol Acyltransferase (CAT) resistance cassette [73] via double homologous recombination using a specific CAT ATS1 KO cosmid [74]. Probes and restriction sites used for Southern blot are indicated by arrowheads and restriction enzymes (RE) names, respectively. (B) Schematic representation of homologous recombination between the CAT KO cosmid and TgATS1 locus. Probe and restriction sites used for Southern blot are indicated by arrowheads and RE names. (C) PCR confirms loss of endogenous copy of eTgATS1 (arrows showing the positive clones). (D) Southern blot analysis of the iTgATS1/ΔTgATS1 clone and its parental iTgATS1/eTgATS1 line confirming eTgATS1 disruption presence of iTgATS1. (TIF) [file ppat.1005765.s002.tif]

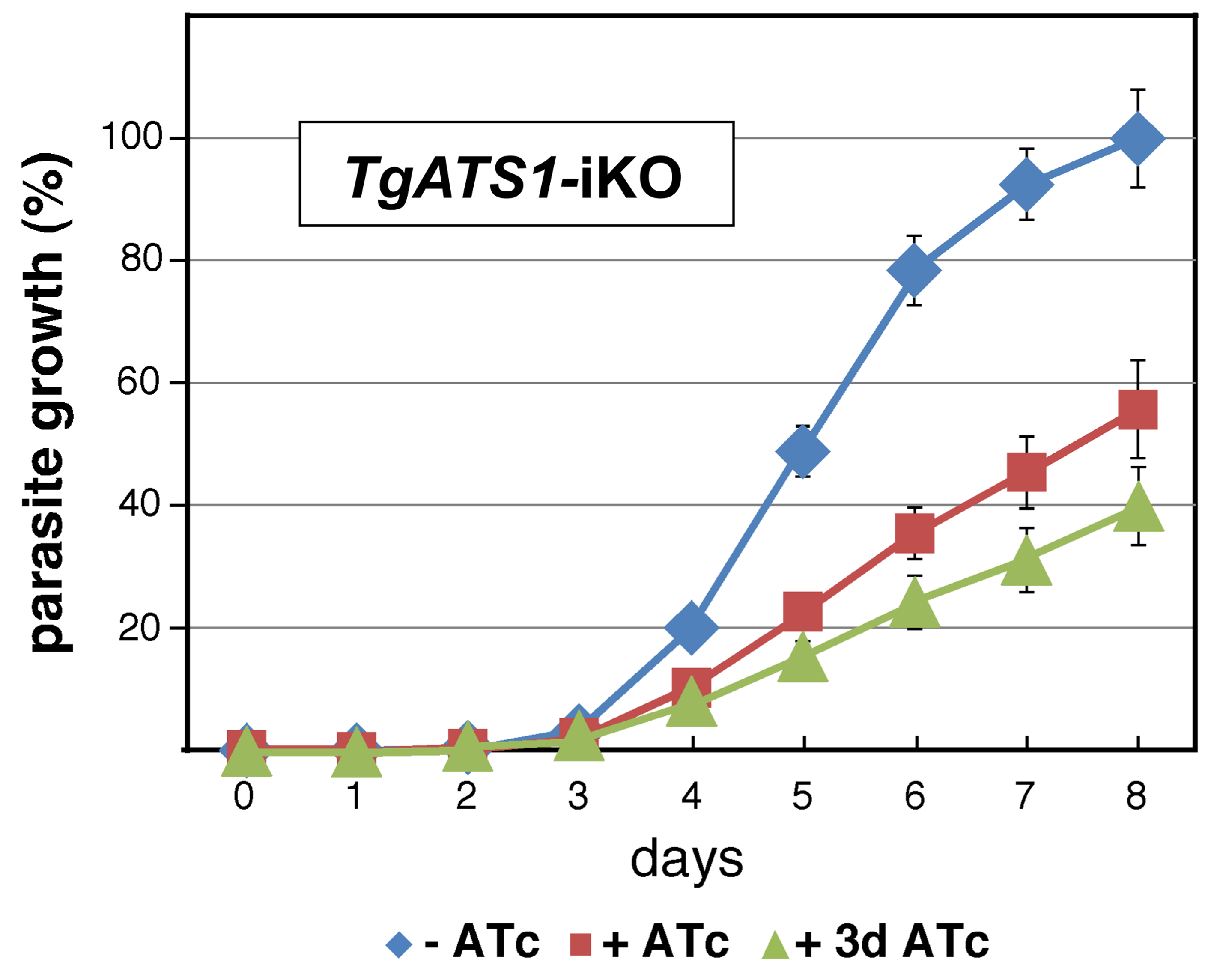

Supplement: S3 Fig — Parasite growth rate was analysed over the course of 8 days by quantifying the fluorescence of tdTomato [71] expressed in the cytosol of TgATS1-iKO parasites in the absence (blue rectangles, control) or the presence of ATc. TgATS1-iKO were grown in the presence of ATc from day 0 to day 8 (red squares) or pre-treated with ATc for 3 days prior to the 8 day ATc treatment (green triangles). In the absence of ATc, TgATS1-iKO grew normally as observed by fluorescence levels but the presence of ATc substantially affected the amount of fluorescence, with this effects being strongest with 3 days of pre-treatment. (TIF) [file ppat.1005765.s003.tif]

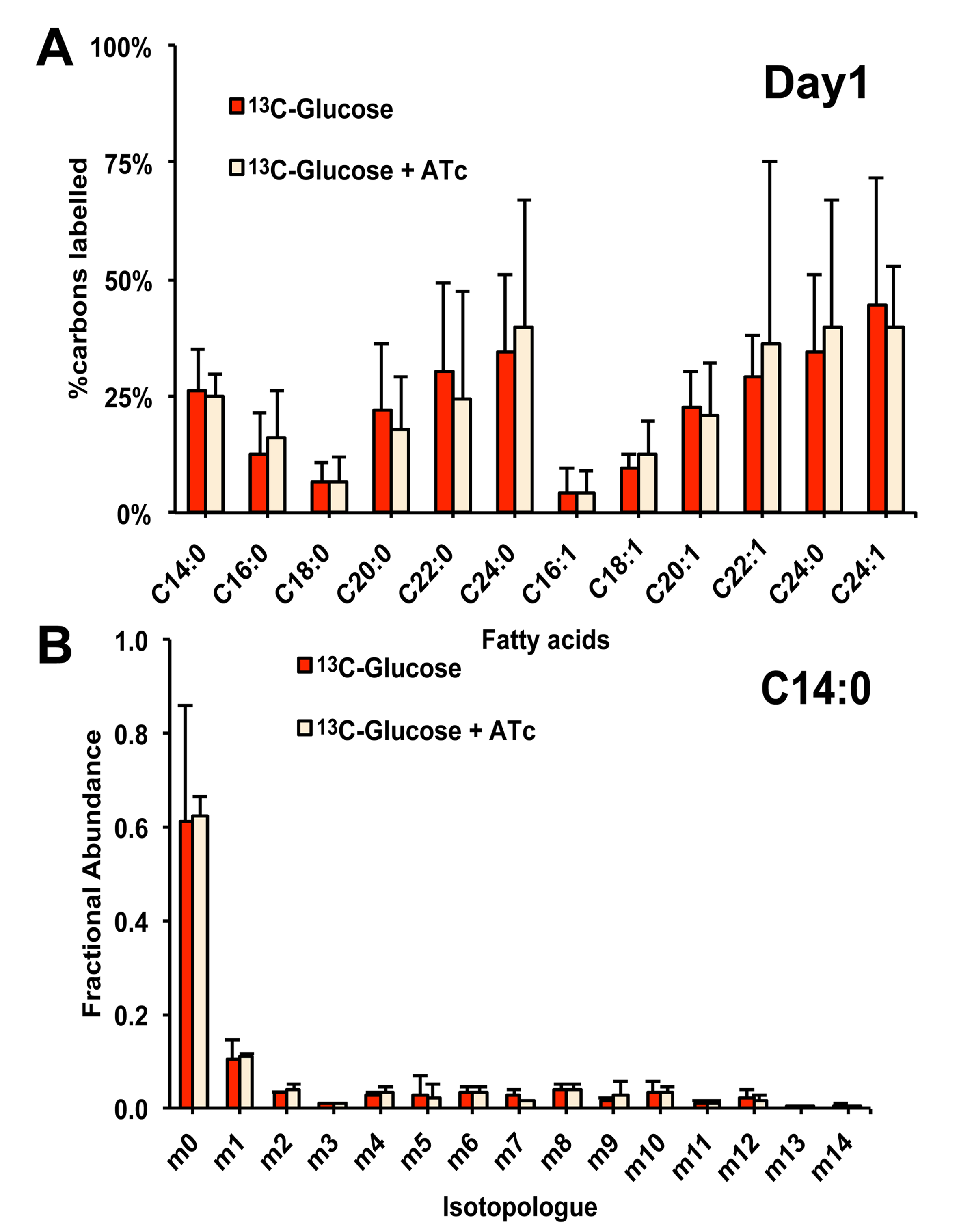

Supplement: S4 Fig — Tachyzoites of T. gondii conditional mutants for ATS1 were labelled with U-13C-glucose for 1 day in the presence or absence of ATc. Lipids were extracted, derivatized, and the resulting FAMEs were analysed by GC-MS to determine 13C incorporation. (A) The mean label incorporation from U-13C-glucose into fatty acids is shown for parasites grown in the absence (dark red) and presence (light red) of ATc. (B) The MIDs for C14:0 labelled with U-13C-glucose in the presence (red) and absence (pale red) of ATc. The x-axis indicates the number of 13C atoms in each FAMEs (‘m0’ indicates the monoisotopic mass containing no 13C atoms, while ‘mX’ represents that mass with ‘X’ 13C atoms incorporated). Nomenclature Cx:y is shown where x is the number of carbons and y is the number of double bonds in the fatty acid chain. Error bars indicate standard deviation, where n = 2 biological replicates. Data shown has been background-subtracted for natural isotope abundance. (TIF) [file ppat.1005765.s004.tif]

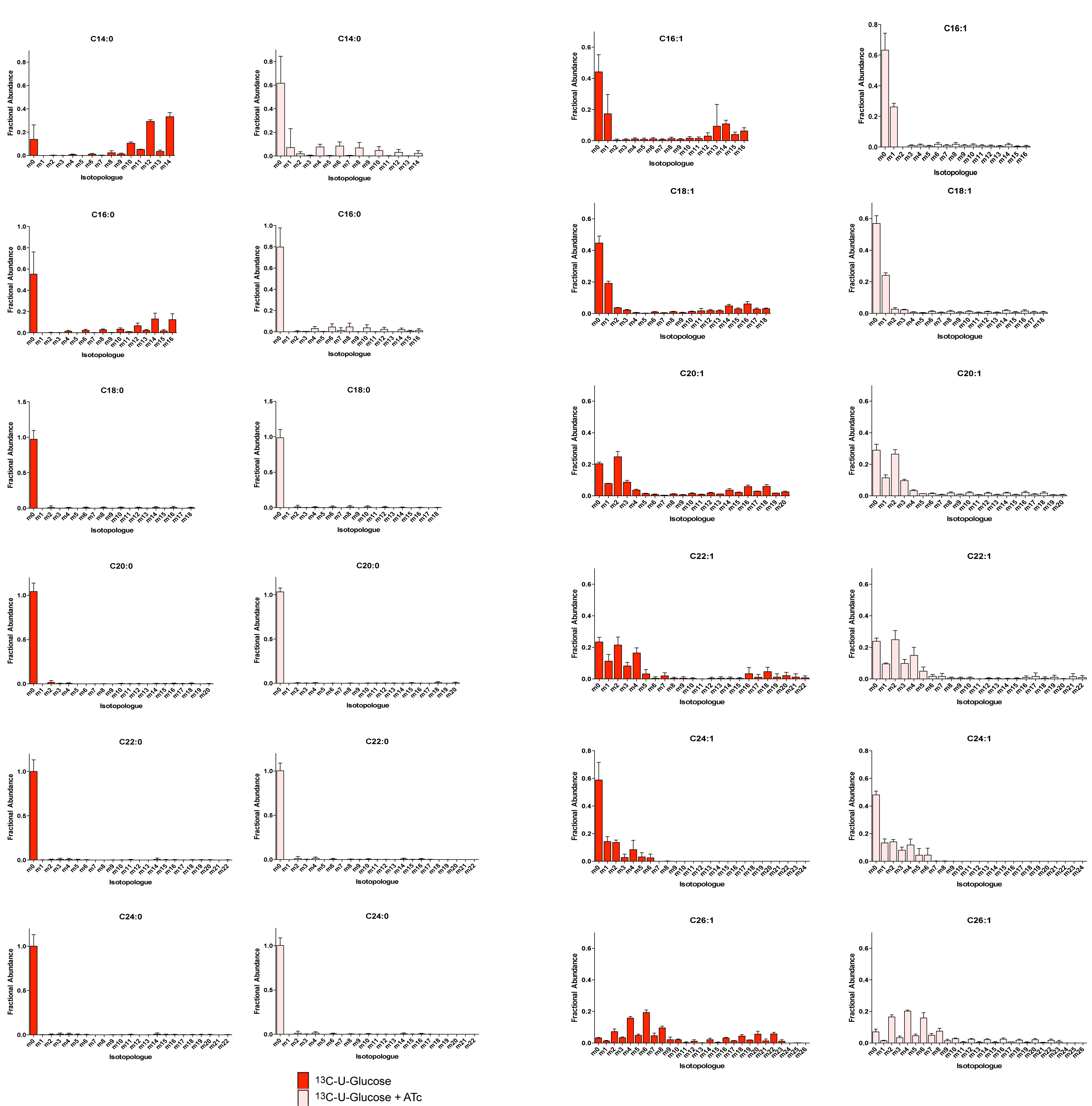

Supplement: S5 Fig — Tachyzoites of T. gondii conditional mutants for TgATS1 were labelled with U-13C-glucose in the presence or absence of ATc. Lipids were extracted, derivatized, and the resulting fatty acid methyl esters (FAMEs) were analysed by GC-MS to determine isotope incorporation. MIDs for all detected FAMEs labelled with U-13C-glucose in the absence and presence of ATc are shown in red and pale red, respectively. The x-axis indicates the number of 13C atoms in each FAMEs (‘m0’ indicates the monoisotopic mass containing no 13C atoms, while ‘mX’ represents that mass with ‘X’ 13C atoms incorporated). Nomenclature Cx:y is shown where x is the number of carbons and y is the number of double bonds in the fatty acid chain. Error bars indicate standard deviation, where n = 4 biological replicates. Data shown has been background-subtracted for natural isotope abundance. (TIF) [file ppat.1005765.s005.tif]

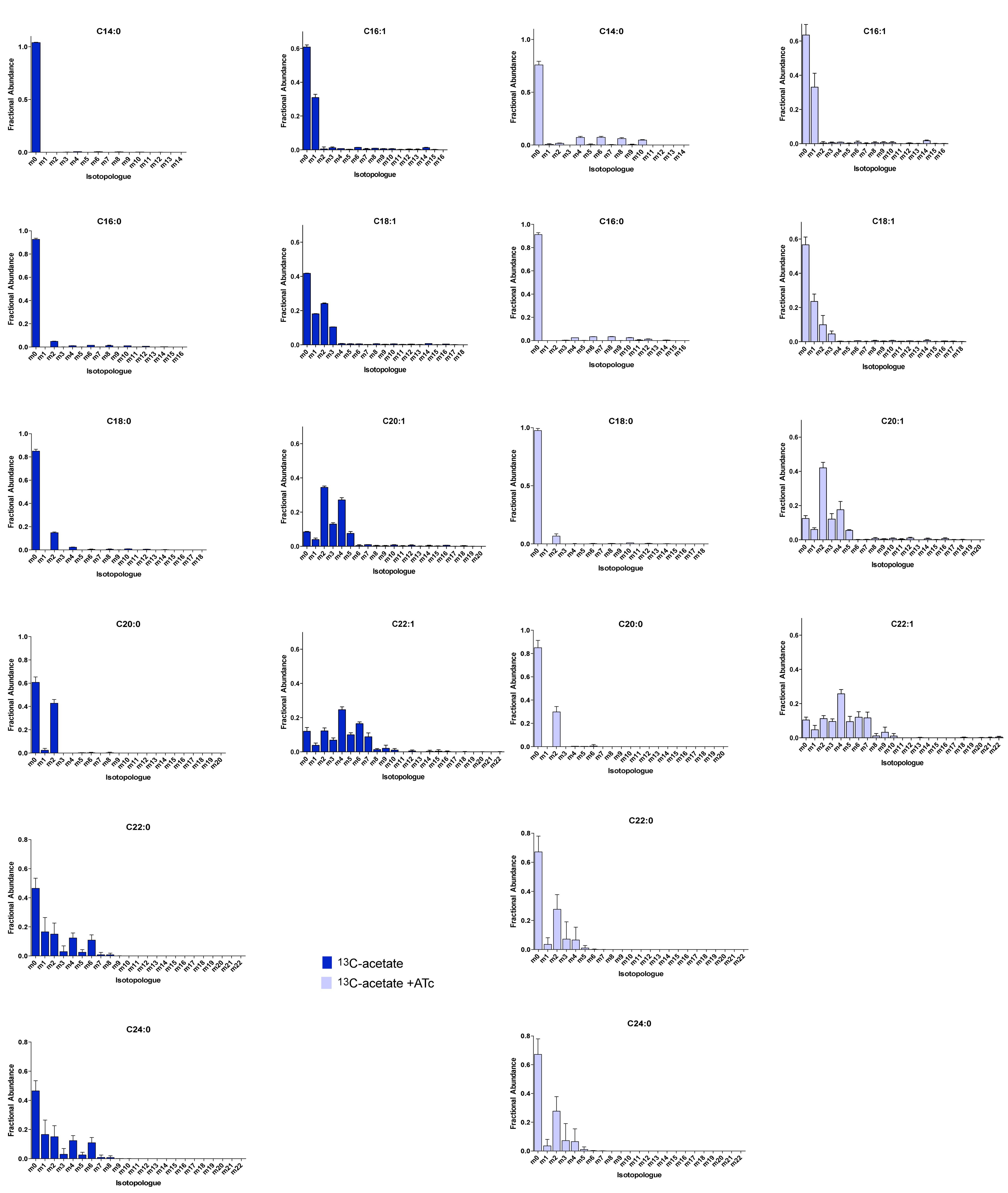

Supplement: S6 Fig — Tachyzoites of TgATS1-iKO parasites were labelled with 13C-U-acetate in the presence or absence of ATc. Lipids were extracted, derivatized, and the resulting FAMEs were analysed by GC-MS to determine isotope incorporation. MIDs for all detected FAMEs labelled with U-13C-acetate in the absence and presence of ATc are shown in purple and pale purple, respectively. MIDs suggested that saturated/monounsaturated FAs incorporated 13C-atoms onto C16:0 or C14:0 in a units of two (i.e. C18 incorporated 2 or 4, C20 incorporated 4 or 6, C22 incorporated 6 or 8, and C24 incorporated 8 or 10 13C-atoms onto C16:0 or C14:0, respectively). The x-axis indicates the number of 13C atoms in each FAME (‘m0’ indicates the monoisotopic mass containing no 13C atoms, while ‘mX’ represents that mass with ‘X’ 13C atoms incorporated). Nomenclature Cx:y is shown where x is the number of carbons and y is the number of double bonds in the fatty acid chain. Error bars indicate standard deviation, where n = 4 biological replicates. Data shown has been background-subtracted for natural isotope abundance. (TIF) [file ppat.1005765.s006.tif]

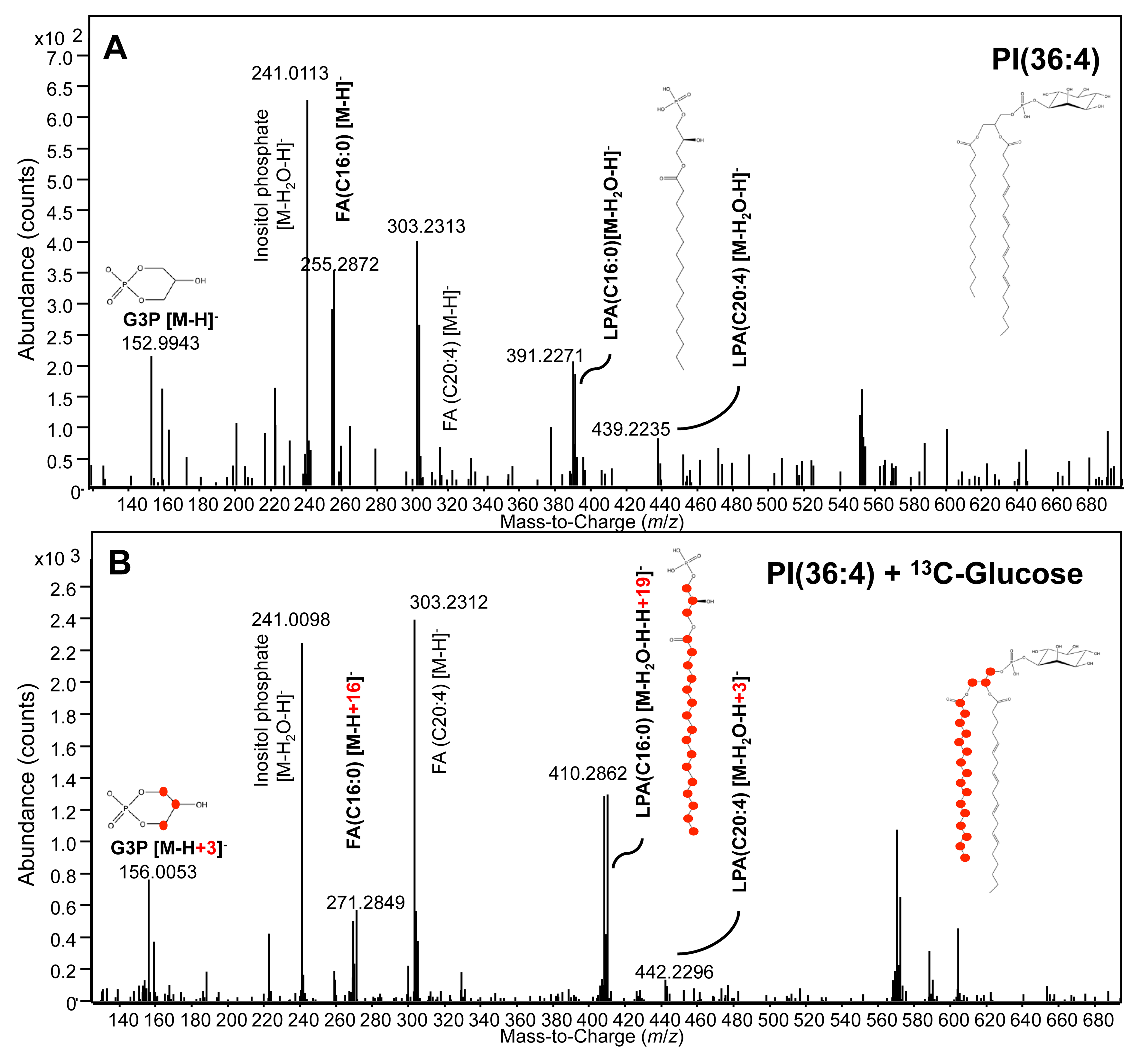

Supplement: S7 Fig — (A) Negative ion mode MS/MS fragmentation of unlabelled PI(36:4), m/z 857.51, extracted from TgATS1-HA-iKO cells grown in the absence of ATc. Characteristic fragment ions were detected as follows: LPA(20:4), m/z 439.22; LPA(16:0), m/z 391.22; FA(C20:4), m/z 303.23; FA(C16:0), m/z 255.28; inositol phosphate, m/z 241.01; glycerol 3-phosphate (G3P), m/z 152.99. (B) Equivalent MS/MS fragmentation of 13C-labelled PI(36:4), m/z 876.5711. Fragment ions corresponding to labelled and unlabelled moieties were observed as follows: LPA(20:4) containing labelled G3P, m/z 442.22; LPA(16:0) containing labelled G3P and labelled C16:0, m/z 410.29; labelled C16:0, m/z 271.28; G3P M+3, m/z 156.00. (TIF) [file ppat.1005765.s007.tif]

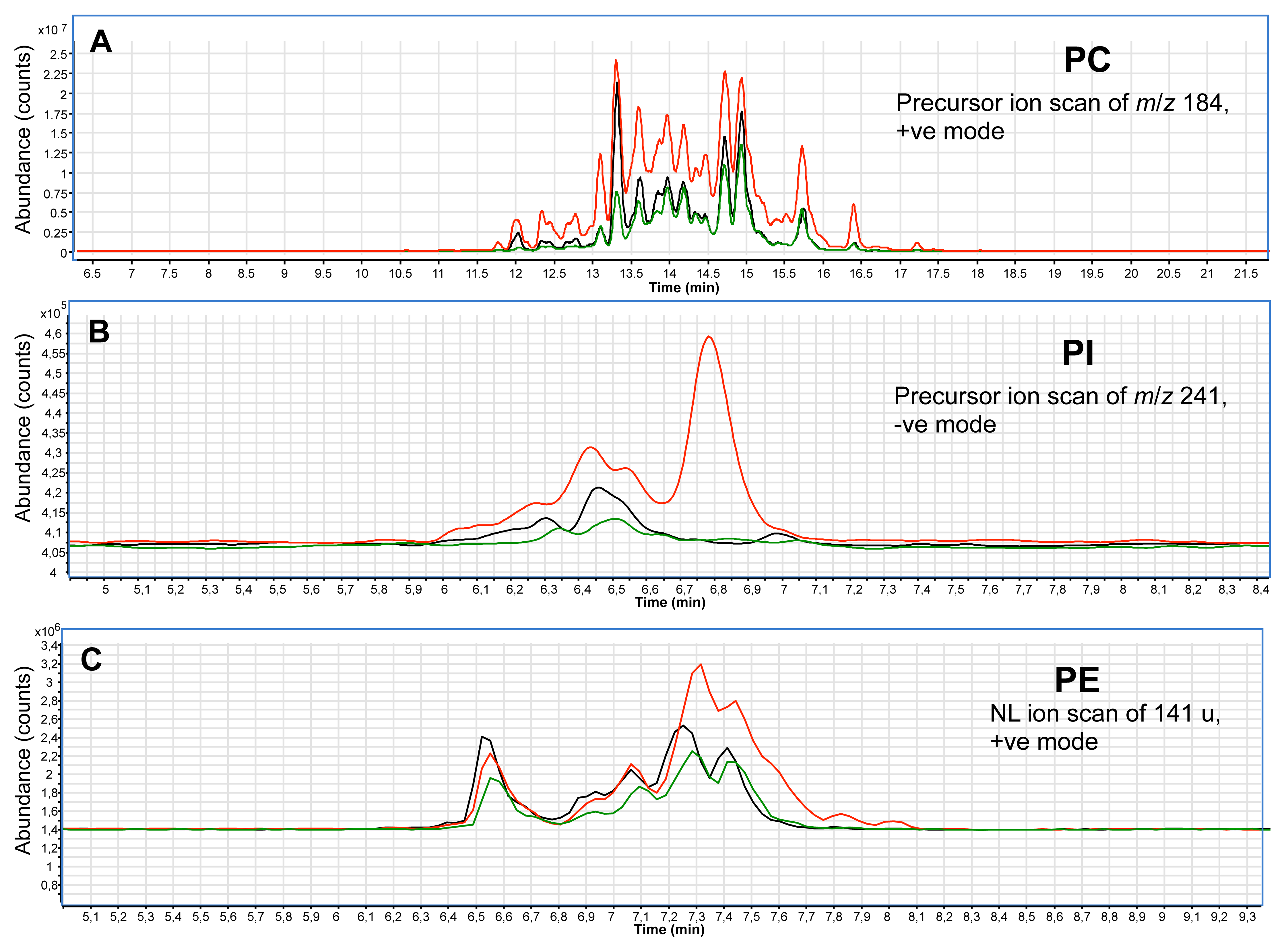

Supplement: S8 Fig — Chromatograms of PC, PE and PI extracted from TgATS1-HA-iKO parasites grown for four days in unlabelled conditions (black line) or 13C-glucose-labelled conditions in the absence (red line) or presence (green line) of ATc. (A) Total ion chromatogram from m/z 184 precursor ion scan in positive mode (for PC). (B) Total ion chromatogram from m/z 241 precursor ion scan in negative mode (for PI). (C) Total ion chromatogram from 141 u neutral loss scan in positive mode (for PE). (TIF) [file ppat.1005765.s008.tif]

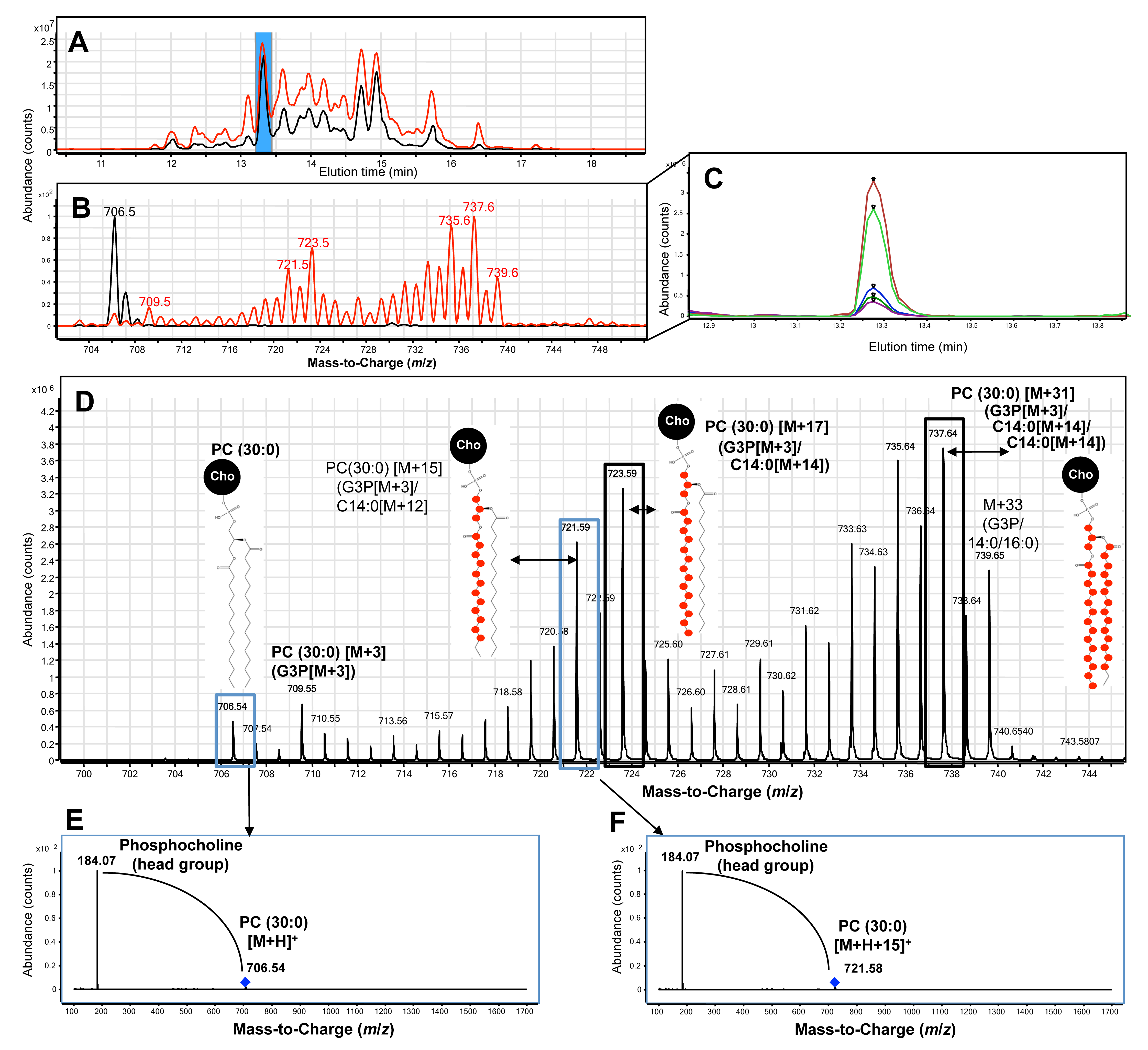

Supplement: S9 Fig — (A) Chromatogram of PC species determined by m/z 184 precursor ion scanning in positive mode for parental strain (black line) and TgATS1-iKO grown with U-13C-glucose in the absence of ATc (red line). PC(30:0) elutes at 13.27 min (blue frame). (B, C) Corresponding mass spectra and extracted ion chromatograms of PC eluting at 13.27 min. (B) A single peak of m/z 706.54 corresponding to PC(30:0) elutes in the parental strain (black line), whereas a series of peaks ranging from m/z 706.54 to 739.6 elute in the U-13C-glucose-labelled TgATS1-iKO (red line). (C) Extracted ion chromatogram of marked ion peaks from (B) all overlap precisely at 13.27 min (arrowheads) and they have similar peak shapes, indicating that they are isotopologues of the same molecule. (D) Putative PC(30:0) isotopologue structures of the multiple 13.27 min ion peaks from labelled TgATS1-iKO as follows: m/z 706.54, unlabelled PC(30:0); m/z 709.56, PC(30:0) containing labelled G3P; m/z 721.59, PC(30:0) containing labelled G3P and C14:0 labelled with 12 13C atoms; m/z 723.59, PC(30:0) containing fully-labelled LPA(14:0); and m/z 737.64 PC(30:0) containing labelled G3P, fully-labelled C14:0 and C16:0 labelled with 14 13C atoms. The m/z 737.64 ion may also represent PC(30:0) containing labelled G3P, fully-labelled C16:0 and C14:0 labelled with 12 13C atoms. (E, F) Representative MS/MS fragmentation of annotated PC molecules. This confirmed that all 13C-labelled molecules observed in the spectrum were PC(30:0) by detection of typical m/z 184 phosphocholine polar head. (TIF) [file ppat.1005765.s009.tif]

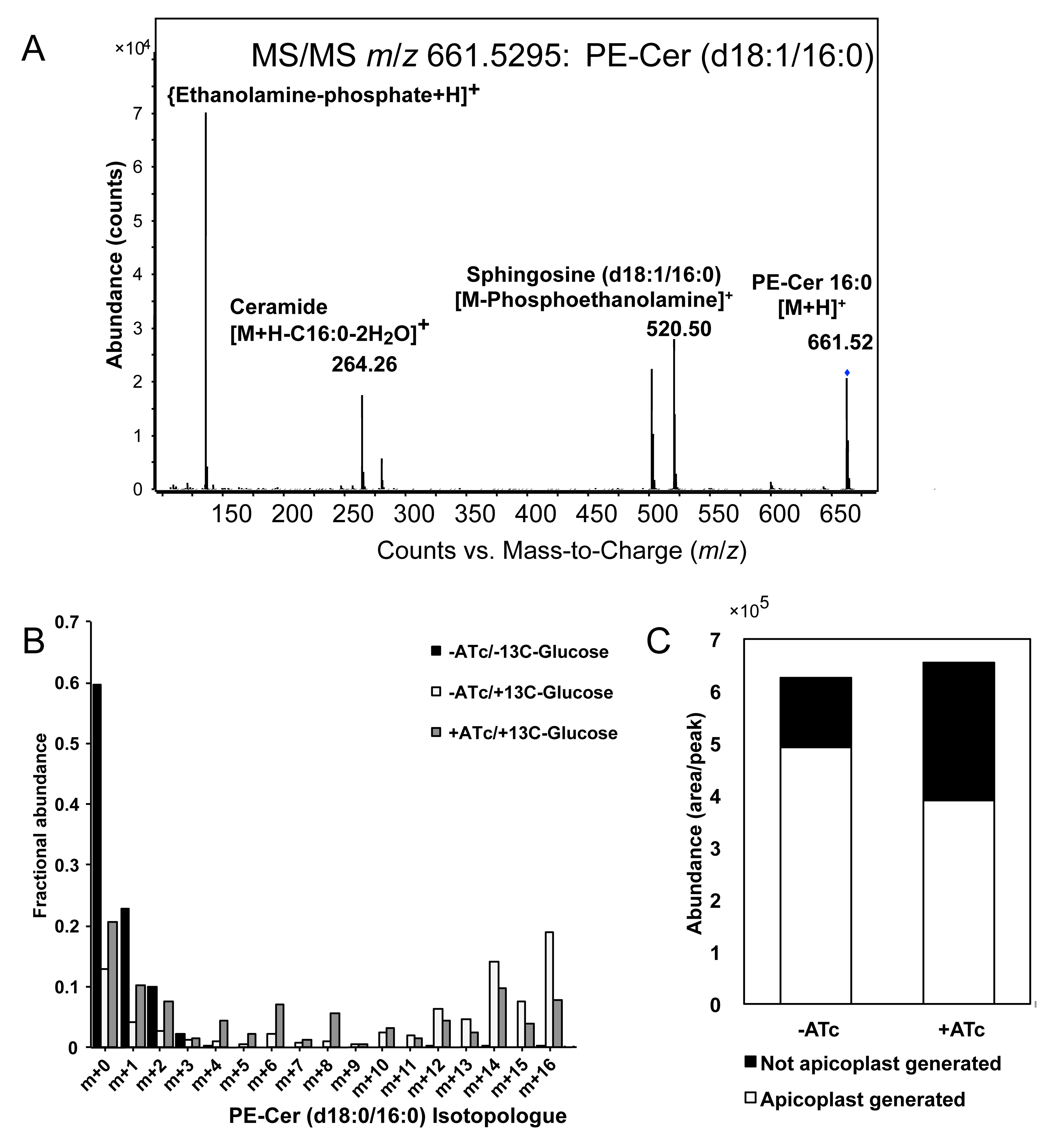

Supplement: S10 Fig — Related to Fig 6: LC-MS/MS structure confirmation of PE-Ceramide(d18:1/16:0) (A) Fragmentation of m/z 661.52 confirmed the assignment of the peak as PE-Cer(d18:1/16:0) by detection of the characteristic ions of ceramide and ethanolamine head group in positive mode: m/z 142 corresponds to ethanol amine phosphate; m/z 520.50 to sphingosine (d18:1/16:0) and m/z 264.2 to ceramide. (B) MIDs of PE-Cer(d18:1/16:0) extracted from TgATS1-iKO parasites grown in unlabelled conditions (black bars) or labelled with U-13C-glucose for 4 days in the absence (white bars) or presence of ATc (grey bars). ‘m0’ indicates the monoisotopic mass containing no 13C atoms, while ‘mX’ represents that mass with ‘X’ 13C atoms incorporated. (C) Relative abundances of apicoplast-generated FA moieties (i.e. those containing 4 or more 13C atoms) in PE-Cer(d18:1/16:0) extracted from TgATS1-iKO parasites grown in the presence and absence of ATc shows that the majority of FA is generated in the apicoplast and was not greatly affected by the disruption of TgATS1. (TIF) [file ppat.1005765.s010.tif]

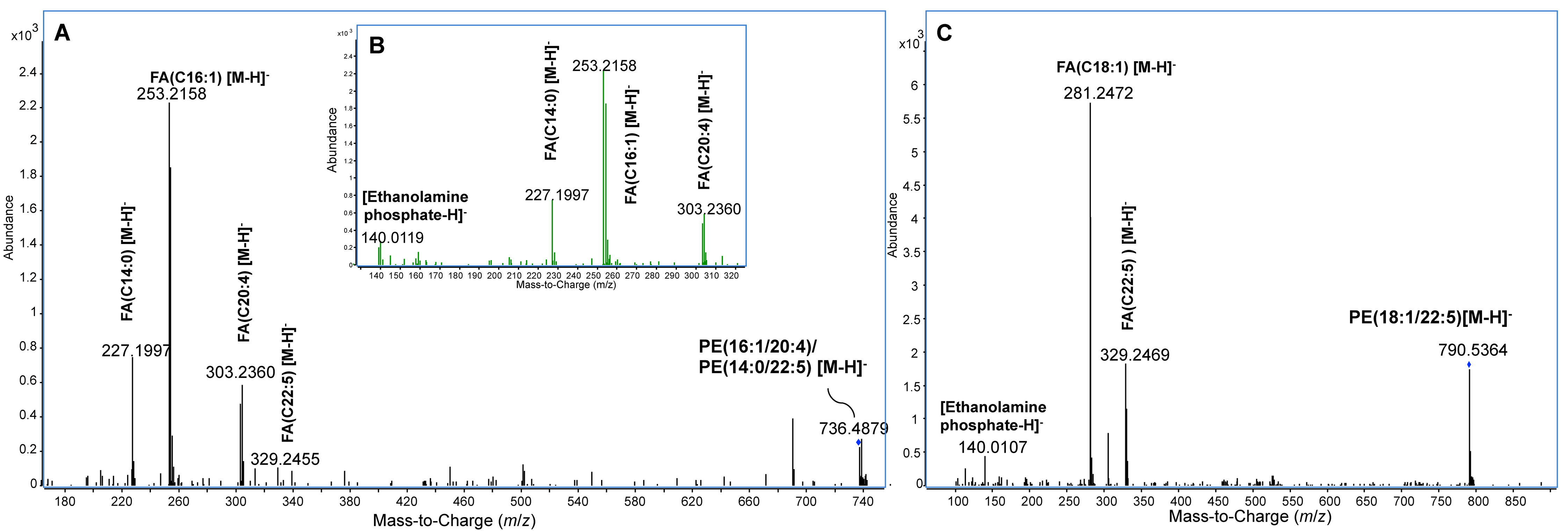

Supplement: S11 Fig — (A) Negative ion mode MS/MS fragmentation of m/z 736.4879 eluting at 13.94 min reveals the presence of ions corresponding to FA(C22:5), m/z 329.24; FA(C20:4), m/z 303.23; FA(C16:1), m/z 253.21; and FA(C14:0), m/z 227.19. (B) Detail from panel A. Presence of a m/z 140.01 ion suggests that this ion could be a mix of PE(16:1/20:4) and PE(14:0/22:5). (C) Negative ion mode MS/MS fragmentation of m/z 790.5364 eluting at 15.16 min revealed the presence of ions corresponding to FA(C22:5), m/z 329.24; FA(C18:1), m/z 281.24; and, potentially, ethanolamine-phosphate, m/z 140.01. This suggests that the mass m/z 790.5364 corresponds to PE(18:1/22:5). (TIF) [file ppat.1005765.s011.tif]
